# Explainable and Interpretable AI for Voice and Speech Analysis in Clinical Care: Systematic Review

**DOI:** 10.2196/83790

**Published:** 2026-06-24

**Authors:** Mohamed Ebraheem, Jamie Toghranegar, Bridge2AI-Voice Consortium, Amanda Chao, Yael Bensoussan, John Michael Templeton

**Affiliations:** 1 Bellini College of Artificial Intelligence, Cybersecurity and Computing, University of South Florida, 4202 East Fowler Avenue, Tampa, FL, 33620, United States, 1 8135850780; 2 Department of Otolaryngology Head and Neck Surgery, USF Health Voice Center, University of South Florida, Tampa, FL, United States; 3 Medical Engineering, College of Engineering, University of South Florida, Tampa, FL, United States

**Keywords:** explainable artificial intelligence, clinical voice analysis, speech biomarkers, deep learning, interpretability, medical decision support, trustworthy AI, artificial intelligence

## Abstract

**Background:**

Driven by recent advances in artificial intelligence (AI), particularly in medicine, audio-based voice and speech biomarkers are increasingly investigated for various medical applications as a complementary or even alternative modality to traditional medical devices. The adoption of deep learning techniques in recent literature is motivated by their superior performance compared to classical machine learning methods. However, ethical and regulatory concerns regarding the black-box nature of these models have limited their integration into clinical workflows. Consequently, explainable artificial intelligence (XAI) has recently been used to address this issue by generating explanations for opaque model outputs. Ideally, medical XAI systems aim to provide human-understandable, clinically grounded explanations essential for enhanced AI trustworthiness and, thereby, facilitate adoption into real-world clinical settings.

**Objective:**

We conduct a systematic literature review of XAI methods applied for explaining deep learning techniques in audio-based voice and speech clinical applications. We aim to identify what XAI methods have been used to explain the decisions of deep learning voice and speech AI systems in health care, as well as XAI-informed insights. Additionally, we aim to contextualize these findings with respect to clinical applicability and stakeholder relevance. Lastly, we identify opportunities and recommendations for future clinical audio XAI design.

**Methods:**

We used PRISMA (Preferred Reporting Items for Systematic Reviews and Meta-Analyses). Six electronic databases (IEEE Xplore, ACM Digital Library, Scopus, PubMed, Web of Science, and Nature) were searched for papers published between January 2015 and February 2025. Eligible studies applied explainability or interpretability methods to deep learning models for voice or speech audio in health care contexts. Risk of bias was assessed using PROBAST+AI (Prediction Model Risk of Bias Assessment Tool). The results were thematically synthesized across explainability categories, input representations, clinical domains, validation strategies, and stakeholder considerations.

**Results:**

A total of 30 studies met the inclusion criteria. These studies used a range of explainability approaches, including gradient-based methods, perturbation-based techniques, surrogate model–based methods, model-internal representation analyses, concept-based detectors, and attention-based explanations. Applications spanned diverse clinical domains, including voice disorders, neurodegenerative diseases, psychiatric conditions, and traumatic brain injury. Overall, results indicate that most studies relied primarily on qualitative interpretation of explainability outputs, with limited quantitative validation of explanation consistency across external datasets. Furthermore, none of the included studies explicitly conducted human-in-the-loop evaluations with relevant stakeholders, highlighting a substantial gap in stakeholder alignment.

**Conclusions:**

Current XAI practices in clinical voice and speech analysis are limited by insufficient validation, lack of domain-specific design, and misalignment with clinical stakeholder needs. This review highlights opportunities for developing validated, audio-aware, and stakeholder-centered XAI approaches to support trustworthy clinical deployment. Interpretation of these findings should consider limitations related to single-reviewer study selection, potential high-risk of bias, and the repeated use of benchmark datasets.

## Introduction

### Overview

Voice is a rich modality that has garnered the interest of the research community for its potential in numerous medical applications [1,2]. Voice and speech biomarkers have recently been used for voice pathology detection [3-5], voice quality assessment [6,7], neurodegenerative disease diagnosis [8-13], mental health monitoring [14,15], cardio-respiratory condition classification [16,17], as well as automatic speech recognition (ASR) for disordered speech [18-20]. The main reason behind this interest in audio-based voice and speech biomarkers is the costliness and invasiveness of traditional voice evaluation techniques, such as laryngoscopy, stroboscopy, laryngeal electromyography, and imaging technologies such as MRI (magnetic resonance imaging) and CT (computed tomography) scans, which limit accessibility for many. Alternatively, artificial intelligence (AI)–driven audio-based medical systems pave the way for broader access to medical services for marginalized and underprivileged populations [21].

Yet, AI integration into real clinical settings is limited, in part due to the scarcity of high-quality data needed to train reliable and fair models [22]. Consequently, myriad large-scale projects are underway for the purpose of collecting extensive and representative voice audio datasets. National Institutes of Health–funded Bridge2AI is a multi-institution, large-scale project that aims to collect standardized, AI-ready, and ethically sourced voice data across various health conditions, where voice and speech samples have been collected from 442 participants [23]. Similarly, AphasiaBank (part of TalkBank Project, Carnegie Mellon University) is another National Institutes of Health–funded endeavor that has amassed multimodal data from 306 persons with aphasia [24]. Launched in 2023 and funded through 2028, SpeechDx is a global initiative dedicated to creating an extensive dataset of Alzheimer’s speech biomarkers and has recruited about 2000 participants [25]. These efforts demonstrate the general recognition of the potential of voice and speech AI in routine clinical practice.

Recently, the trustworthiness of AI systems has been a central issue for clinical integration of AI, particularly the obscurity of the decision-making processes of deep learning models to end users [22,26-29]. Known as the “black-box” problem, this issue is especially critical in medicine, where decisions directly impact patient safety. Yet, regulatory frameworks have struggled to keep pace with AI’s rapid development, leaving unresolved questions of liability and accountability [30]. Clinicians and regulators must understand how models operate, how reliable they are, and under what conditions they fail, before AI systems are integrated into clinical workflows in a meaningful way [31]. Only then can patients be guaranteed safe, high-quality medical care. On one end, there are opinions against the use of black-box models at all for high-stakes environments such as medicine [32]. While white-box, classical machine learning (ML) models such as decision trees or support vector machines trained on interpretable, hand-crafted features offer greater transparency, deep learning models consistently achieve superior performance; this is known as the interpretability-accuracy trade-off [33].

Explainable artificial intelligence (XAI) has emerged to ameliorate this challenge, aiming to make black-box models more transparent [22,29,31,34]. However, many XAI techniques have been developed for image-based domains, such as overlaying saliency maps on brain MRI or chest CT scans. In voice analysis, raw audio is often transformed into time-frequency representations such as spectrograms before being input into deep learning models. Mapping a region on a spectrogram back to an intuitively understandable auditory event (eg, a tremor on a specific syllable) is inherently more complex than identifying a visible tumor on an x-ray. The relationship between spectral features and perceived voice quality or pathology is often highly nonlinear, making clinical interpretation challenging.

Furthermore, the intuitiveness of model explanations varies substantially with respect to end users [35-37]. Computer scientists and ML researchers may be able to interpret technical visualizations such as activation maps or feature attribution plots, whereas clinicians, patients, and regulators have different knowledge bases, priorities, and constraints. A “one-size-fits-all” approach to XAI design is therefore inappropriate, especially when explainability is positioned as a pathway to increasing trust. Thereby, multidisciplinary effort in the design of XAI methods is vital for providing appropriate presentations of explanations suited for the diverse backgrounds and needs of the respective stakeholders [37,38].

In this context, this paper presents a systematic literature review of XAI methods applied to deep learning models for clinical voice and speech analysis. In this review, we use “clinical audio” and “clinical voice and speech” to refer to voice or speech data derived from individuals with a clinical condition used for a health care–relevant application or outcome, regardless of whether the recording occurred in a controlled clinical environment. The review addresses the following research questions:

- What XAI methods have been used to explain the decisions of deep learning voice and speech AI systems in health care?
- What insights are derived from the application of these XAI methods?
- What are the limitations of these XAI methods in the context of clinical audio in terms of clinical applicability and stakeholder relevance?

The remainder of this paper is organized as follows. The rest of the Introduction section presents background for XAI concepts and different perspectives regarding the definitions of explainability and interpretability. We also outline the objectives of XAI and provide a discussion on the broad application of XAI in diverse medical domains. The Methods section describes the systematic review methodology. The Results section presents explainability approaches, explainability validation strategies, and human-centered evaluation. Then, we discuss the limitations of current approaches, stakeholder alignment, and the impact of audio representation on interpretability, upon which future directions are presented.

### Background

#### Explainability vs Interpretability

There is no clear consensus in the literature on the definitions of explainability and interpretability in the context of AI, and the two terms are often used interchangeably. Nonetheless, various works have attempted to provide distinctions between them.

Linardatos et al [39] highlight the persistent ambiguity surrounding these concepts and review the different ways they have been differentiated in literature. They describe interpretability as relating to the intuition behind a model’s outputs, such that a more interpretable model makes it easier to identify causal relationships between inputs and outputs. On the other hand, explainability is concerned with the internal logic and mechanics of the model. They conclude that interpretability is the broader term, and that a model can be interpretable without being explainable.

Gilpin et al [40] approach the distinction differently, defining explainability as a means of answering the questions “why” or “why not” a system behaves in a particular way. In contrast, they describe interpretability as the ability to represent the model’s internal processes in a human-understandable form, emphasizing that this is dependent on the knowledge and needs of the target user.

Das and Rad [41] define interpretability as a quality of a system in which its expressions convey human-understandable insights into how it works. They differentiate explanations as additional metadata—produced either by the model or an external algorithm—that clarify the relationship between inputs and outputs.

The National Institute of Standards and Technology also provides guidance on explainability, outlining four principles for explainable systems, the most important of which is that they should deliver accompanying justifications or reasons for model outputs [42]. The work further distinguishes between self-interpretable models, which are inherently understandable to humans, and post hoc explanations, which are generated by an explainability algorithm to provide insight into otherwise opaque models.

In summary, interpretability relates to the inherent transparency of the model itself; white-box models, such as decision trees and linear regression, are generally considered interpretable, while explainability refers to the generation of additional information (ie, explanations) that clarify the reasoning behind a model’s decision, regardless of the model’s inherent transparency. In this work, we adopt the latter definition.

#### Objectives of Explainable Artificial Intelligence

##### Overview

XAI serves multiple partially overlapping objectives, which vary according to the target application and the perspective of the stakeholder [43,44]. The following categories are directly derived from other studies [43,44].

##### Debugging and Monitoring

Explanations help AI developers identify model errors, biases, and spurious correlations, thereby revealing opportunities for performance improvement. XAI can also be used to monitor performance drift during deployment, ensuring the model continues to operate as intended over time.

##### Evaluation and Validation

XAI enables stakeholders to assess whether a model is appropriate, reliable, and clinically valid for a given application. It supports both predeployment evaluation and ongoing validation, ensuring that model decisions remain aligned with intended clinical outcomes.

##### Justification and Transparency

XAI fosters trust among stakeholders by providing context-relevant rationales for model decisions. This includes justifying individual predictions and improving transparency in the decision-making process, allowing clinicians, patients, and regulators to audit and verify the system’s outputs.

##### Improvement and Learning

Explanations support iterative model refinement through collaboration with domain experts, enhancing alignment with clinical reasoning. XAI can also contribute to the discovery of new domain knowledge, such as identifying previously unknown biomarkers.

##### Governance and Compliance

In high-stakes domains such as medicine, XAI facilitates compliance with ethical, legal, and regulatory requirements, including provisions such as the European General Data Protection Regulation “right to explanation” [45,46]. It enables model auditability, supports liability attribution, and strengthens governance processes.

### Characteristics of XAI Methods

#### Overview

Traditionally, XAI methods are categorized according to their scope of application (ie, model-specific and model-agnostic). Classical taxonomy groups XAI methods into intrinsic methods, where the model contains native interpretable or explainable components, and post hoc techniques, where a secondary system generates explanations. The resultant explanations provide either local insight for individual sample instances or globally justify model behavior. Accordingly, methods in the literature are categorized in the Results section (Table 1).

**Table 1.** Summary of XAI^a^ methods reported in the included studies, classified according to model dependency (model-agnostic vs model-specific), scope of explanation (local vs global), relationship to the predictive model (post hoc vs intrinsic), and explanation modality. The table shows ablation analysis and latent space interpretation dominating the literature, along with Grad-CAM^b^.

XAI methods	Model agnostic/specific	Global/local	Post hoc/intrinsic	Explanation modality	Implemented in
Grad-CAM	Specific	Local	Post hoc	Visual	[3,6,10,47-49]
Guided backpropagation	Specific	Local	Post hoc	Visual	[50]
Saliency maps	Specific	Local	Post hoc	Visual	[4]
Eigen-CAM^c^	Specific	Local	Post hoc	Visual	[10]
SHAP^d^	Agnostic	Local	Post hoc	Tabular	[9,51,52]
GradientSHAP^e^	Specific	Local	Post hoc	Tabular/visual	[53]
LIME^f^	Agnostic	Local	Post hoc	Tabular/visual	[9]
xDMFCC^g^	Agnostic	Local	Post hoc	Visual/tabular	[54]
Ablation studies	Specific	Global	Post hoc	Tabular	[11,12,14,15,18,20,55,56]
Simple attention	Specific	Local	Intrinsic	Visual	[52]
Attention rollout	Specific	Local	Post hoc	Visual	[57]
Concept detectors network	Specific	Global	Intrinsic	Concept-level	[7,58]
Sinc filters	Specific	Global	Intrinsic	Conceptual (filter shape)	[5,53]
Feature map analysis	Specific	Global	Post hoc	Visual (CNN^h^ filters)	[13,20]
t-SNE^i^	Agnostic	Global	Post hoc	Visual	[3,5,19,20,49,56,57,59,60]

a XAI: explainable artificial intelligence.

b Grad-CAM: gradient-weighted class activation mapping.

c Eigen-CAM: Eigen class activation mapping.

d SHAP: Shapley Additive Explanations.

e GradientSHAP: gradient Shapley Additive Explanations.

f LIME: local interpretable model-agnostic explanation.

g xDMFCC: explainable deep learning mel-frequency cepstral coefficients.

h CNN: convolutional neural network.

i t-SNE: t-distributed stochastic neighbor embedding.

#### Model-Specific vs Model-Agnostic

Model-specific methods exploit structural aspects particular to model type or architecture to, in theory, produce higher-quality explanations. For example, Grad-CAM (gradient-weighted class activation mapping) is applicable to convolutional neural networks (CNNs) and takes advantage of their spatial feature maps to highlight input features that are most influential for the output.

In contrast, model-agnostic methods, such as SHAP (Shapley Additive Explanations), are generalizable to any ML model regardless of architecture. This universality enables versatile implementation across many applications. Unlike model-specific techniques that benefit from internal model architecture, model-agnostic methods often exhibit lower fidelity and efficiency, especially when dealing with high-dimensional data.

#### Local vs Global

Local explainability methods aim to clarify the reasoning behind a model’s prediction for a single input instance. These explanations are instance-specific and do not describe the model’s overall decision-making process. For example, visualization techniques such as Class Activation Maps can generate heatmaps that indicate the regions of an input spectrogram most relevant to the model’s prediction. Local explanations are particularly valuable in clinical decision support scenarios where individual case justification is critical.

Global explainability methods, on the other hand, aim to describe the model’s general behavior across the entire dataset. This can involve identifying the most influential features for distinguishing between classes, mapping decision boundaries, or summarizing feature interactions. Such methods are crucial for understanding systematic model biases, validating clinical relevance, and ensuring that the model’s logic aligns with domain knowledge.

#### Intrinsic vs Post Hoc

As mentioned earlier, intrinsic (or self-) interpretability refers to the degree to which a model’s decision-making process is transparent and human-understandable by design. White-box models are examples of intrinsically interpretable models. However, they often underperform compared to more complex black-box architectures such as deep neural networks.

Post hoc methods, in contrast, are applied after the training process is completed to explain the model’s behavior without altering its internal mechanics. These techniques, ranging from saliency maps to perturbation-based analyses, are particularly prevalent for explaining black-box models.

### XAI in Medicine

With recent advances in medical AI, the implementation of XAI has become increasingly critical to ensure that AI models are reliable, ethical, legally compliant, and clinically aligned, particularly in high-stakes environments such as health care. The literature contains numerous surveys discussing the role of XAI across diverse medical applications.

Several recent reviews have broadly examined the adoption of XAI in medicine, emphasizing its potential to improve transparency, foster trust, and facilitate regulatory compliance [61-65]. These works cover a variety of data modalities, including medical imaging, electronic health records (EHR), genomics, and time-series analysis. Commonly reported methods include model-agnostic techniques, most notably LIME (local interpretable model-agnostic explanations) and SHAP, alongside post hoc visualization approaches. Nonetheless, these reviews consistently highlight persistent challenges relating to faithfulness, stability, and standardized evaluation of explanations.

Domain-specific surveys further illustrate these trends. For example, van der Velden et al [66] reviewed over 200 studies applying XAI to medical imaging, noting the predominance of visualization techniques, followed by perturbation-based, textual, and example-based approaches. While visual explanations have been extensively validated, the authors stress the need for equivalent validation of textual and example-based methods. Muhammad and Bendechache [67] similarly highlight the interpretive ambiguity and perturbation sensitivity of visual methods. In the context of medical time-series data, Caterson et al [68] present a scoping review of XAI for EHR, finding feature attribution methods to be the most widely used. Salih et al [69] review XAI in cardiology for ECG (electrocardiogram) and EHR, reporting the frequent use of SHAP and Grad-CAM, followed by LIME, but also note that nearly half (47%) of the studies did not use any formal evaluation of the explanations produced. In the mental health and psychiatry domain, Joyce et al [70] reviewed XAI applied to neuroimaging, interview transcripts, and physiological data, underscoring the importance of human-centered design for producing clinically useful explanations beyond raw saliency maps. With respect to clinical audio, Chen et al [16] reviewed XAI approaches for vocal biomarker-based lung disease detection, discussing several issues, including the utility of explanations defined in terms of informativeness and user understanding as being an important criterion for evaluating explanations.

Despite the breadth of these surveys, to the best of our knowledge, no systematic reviews have comprehensively examined the use of XAI for clinical voice and speech biomarkers more broadly. This gap highlights the need for a dedicated synthesis in this domain, particularly given the unique challenges and interpretive demands of audio-based clinical decision support systems.

### Unique Challenges of Clinical Audio for XAI

#### Audio Abstraction and Representation

In the time domain, audio is represented as waveforms that fully capture the acoustic signal but are difficult for humans to visually interpret directly. Consequently, audio is often transformed into spectrograms or mel-frequency cepstral coefficients (MFCCs). While clinicians such as speech-language pathologists and audiologists are trained to interpret spectrograms, visual explanations based on time-frequency representations remain suboptimal, particularly for other stakeholder groups. This issue is further highlighted when explanations are inconsistent across samples or vary substantially between different explainability methods, undermining their reliability and interpretive stability [71,72]. This can be attributed to human auditory perception of sound, rather than of the visual [73]. MFCCs, a compact nonlinear “spectrum-of-the-spectrum” representation of audio, are even more obscure to clinicians, limiting their clinical interpretability [47,74]. Additionally, speaker characteristics are usually spread across multiple frequency bands, making the problem of localizing relevant frequency information too broad to solve effectively with traditional vision-based XAI methods [75]. Nonetheless, assuming relevant spatiotemporal regions are identifiable through traditional vision-based methods, understanding why these regions are important remains obscure, at least partially, and requires further analysis to answer such questions. Ultimately, the nonvisual perceptual nature of voice and speech makes current visual explanations a nonideal solution to the overarching problem of explainability and trustworthy clinical AI.

#### Temporal Dynamics

Unlike medical images, where both axes carry spatial meaning, audio has an inherently temporal structure. Identifying when a clinically relevant event occurs (eg, a stutter or pause) is as important as identifying which acoustic features are involved. This mismatch makes it difficult for visualization-based methods such as saliency maps or Grad-CAM, designed for spatial data, to yield clinically actionable explanations in audio [73,75].

#### Annotation Scarcity

Phonetic, prosodic, disfluency, and voice quality annotations are essential for aligning model explanations with known biomarkers; however, such granular annotations, typically performed by multiple trained experts, are resource-intensive and, consequently, scarce. Without such annotations, validation of model explanations’ alignment to medically grounded features becomes increasingly daunting, limiting their utility in practice.

In summary, these challenges highlight that explainability methods developed for domains such as imaging or EHR are not directly transferable to clinical audio. Audio-native evaluation frameworks and human-centered approaches are needed to ensure that XAI methods drive actionable clinical insights.

## Methods

### Systematic Review Search Strategy

We conducted our systematic review according to the PRISMA (Preferred Reporting Items for Systematic Reviews and Meta-Analyses) guideline. A review protocol was prepared, but not registered before this study. Our goal was to survey papers that perform explainability or interpretability analyses of deep learning models applied to clinical voice and speech audio. A comprehensive search was carried out across IEEE Xplore, ACM, Scopus, PubMed, Web of Science, and Nature.

The following search terms were selected across three domains: explainability and interpretability, voice, speech, and acoustics, and clinical and health care context. These terms were used to create a query, which was adapted for each database. The exact search strings for each database can be found in Multimedia Appendix 1. The search was conducted across all databases on February 7, 2025, except ACM, which was searched on February 11, 2025. The search included studies published between January 1, 2015, and the date of the search. In total, 1426 records were retrieved, which were reduced to 1348 after removing 78 duplicates.

### Eligibility Criteria

Papers were selected if they met the following criteria of being (1) focused on deep learning models applied to voice and/or speech data for health care applications, (2) applied explainability or interpretability techniques to the model, (3) reported empirical results or experimental validation, and (4) published in peer-reviewed journals or conferences.

Papers were excluded for any of the following reasons: (1) explainability and/or interpretability analysis performed for non–deep learning models; (2) used audio transcripts only (ie, purely natural language processing methods); (3) used nonclinical audio datasets; (4) not published in English; and (5) reviews, theses, or non–peer-reviewed papers.

### Study Selection

The results of the search strategy were exported to the RAYYAN tool [76] for screening. One reviewer conducted the initial screening of titles and abstracts against the eligibility criteria, and a second reviewer independently verified the filtering decisions. Papers were excluded only if there was clear evidence in the title or abstract that the paper did not meet the criteria; otherwise, they proceeded to full-text review. The screening process resulted in 187 papers deferred for full-text review, of which 2 papers were retracted, and 2 papers were inaccessible. The final set for full-text review was 183 papers.

Full-text screening was performed by two reviewers, with deliberation and discussion used to resolve disagreements. Reasons for exclusion at this stage were documented. The final included set consisted of 30 papers that met all eligibility criteria.

### Data Extraction

Data extraction was carried out by a single reviewer using a standardized spreadsheet. A second reviewer reviewed the extracted data and deliberated on any uncertainties or discrepancies to ensure accuracy and completeness. The following information was extracted from the final set of included papers: (1) bibliographic information (authors, and year), (2) dataset information (clinical condition, number of subjects, and acoustic tasks), (3) clinical application, (4) deep learning methodology (model, hyperparameters, and training or validation strategy), (5) model performance, (6) explainability or interpretability strategy, (7) insights gained from explainability or interpretability analysis, and (8) validation or support for the explainability or interpretability results.

### Data Synthesis

#### Overview

Data were synthesized across explainability method type, explainability input-output characteristics, explainability validation strategies, and stakeholder involvement.

#### Explainability Methods

As existing surveys of XAI demonstrate a lack of consensus on a unified XAI taxonomy, we adopted a set of commonly used XAI method categories reported across prior surveys and taxonomy literature, including work in medical XAI [77-79]. Our work does not aim to propose a new categorization or taxonomy, as this is outside the scope of this study. Accordingly, explainability methods in the included studies were grouped using the following widely adopted categories:

- Gradient saliency-based methods: these methods derive explanations by analyzing gradients of the model output with respect to the input or intermediate feature maps, characterizing the influence of different parts of the input signal on model predictions.
- Perturbation-based techniques: these methods generate explanations by systematically modifying, masking, or removing parts of the input or feature space and observing the resulting change in model output.
- Surrogate model-based methods: this category includes methods that use simpler models to approximate the local behavior of complex models.
- Model-internal representation analysis: this category encompasses methods that provide explainability through inspection and/or visualization of a model’s internal structures.
- Concept-based methods: these methods include techniques that provide explanations by relating model behavior to predefined, higher-level, semantically human-understandable concepts or clinically meaningful attributes.
- Attention-based explanations: this family of methods encompasses techniques that rely on attention weights to generate explanations.

#### Explainability Input-Output Representation

For each included study, we recorded the input representation over which explainability was applied and the corresponding form of the explainability output, as these factors directly determine the interpretability and modality of explanations in clinical audio systems. Input representation refers to the signal or feature representation interrogated by the explainability method (which may differ from that used by the predictive model), while output characteristics refer to how explanations were presented (eg, explanation modality). These attributes were extracted to support the synthesis of explainability practices across studies and to contextualize differences in explanation form, granularity, and clinical interpretability.

#### Explainability Validation Strategies

In this work, an explainability validation strategy is defined as any technical, model-centric procedure used to assess the faithfulness, consistency, or robustness of explainability outputs with respect to the underlying model behavior. This definition explicitly refers to model-centered validation and excludes human judgment or interpretive assessment.

The following explainability validation strategies were recorded for the included studies:

- Perturbation-based validation: input features, time segments, or frequency regions identified as salient by the explainability method are systematically modified, masked, or removed to assess the resulting impact on model predictions or performance.
- Ground-truth or annotation-based verification: salient regions or features identified by the explainability method are compared against externally defined references, such as expert annotations, labeled phonetic events, disorder-specific acoustic markers, or task-related temporal boundaries, when used to evaluate correspondence with model behavior.
- Stability or cross-dataset consistency analysis: explanation patterns are examined across different datasets, cohorts, recording conditions, or evaluation splits to assess the robustness and consistency of explanations under data variation or dataset shift.

#### Domain-Specific Explanation Patterns

To support structured synthesis of explainability findings, study-derived insights were grouped and analyzed according to the clinical application domain targeted by each study (as defined by each study’s objective). Explainability outputs were examined within each domain to identify recurring explanation patterns. We also noted potential risk of circular validation, particularly in cases where multiple studies relied on the same dataset, which may limit the generalizability of observed explanation trends.

#### Human-Centered Analysis and Stakeholder Alignment

Beyond technical explainability validation, human-centered evaluation is critical for assessing explanation quality, the clinical relevance, and practical utility of explainability methods in real-world health care settings. Accordingly, stakeholder alignment was assessed separately from technical validation.

Stakeholder alignment was defined as explicit involvement of domain experts, such as clinicians, speech-language pathologists, or regulatory stakeholders, in interpreting, evaluating, or providing feedback on explainability outputs. For each included study, the presence or absence of human-in-the-loop evaluation was recorded, along with the reported form of involvement.

### Risk of Bias and Applicability Assessment

A formal quality and risk-of-bias assessment was conducted to evaluate the methodological robustness of prediction models estimating health-related outcomes. PROBAST+AI (Prediction Model Risk of Bias Assessment Tool for Artificial Intelligence) [80], an extension of the original PROBAST [81] framework developed to address advances in AI- and ML-based prediction models, was used for this purpose.

A review-level PICOTS (Population; Index Model; Comparator; Outcome; Timing; Setting) framework was predefined to guide the scope and intended applicability of the assessment. The tool was applied to studies that developed or evaluated prediction models estimating health-related outcomes. Studies that did not fit these criteria were not suited for quality assessment by the PROBAST+AI tool. Each eligible study was classified as model development, model evaluation, or both. In accordance with PROBAST+AI guidance, development and evaluation components were assessed separately.

The tool evaluates four domains: (1) participants and data sources: addressing data origin, collection procedures, and representativeness; (2) predictors: examining input definition, preprocessing, and availability at the time of intended use; (3) outcome: assessing outcome definition, measurement, and timing; and (4) analysis: evaluating sample size adequacy, validation strategy, handling of missing data, risk of data leakage, and mitigation of overfitting.

Domains were rated as low, high, or unclear for development (quality concern) and evaluation (risk of bias), respectively. Applicability was assessed for the first three domains only and not for the analysis domain. The fourth domain does not include applicability considerations under the PROBAST+AI framework, as applicability refers to the assessor’s review question or intended use of a model, including the target population and setting. In accordance with PROBAST+AI guidance, if at least one domain was rated high, the overall judgment was classified as high. Detailed signaling criteria are described in Multimedia Appendix 2 [3-6,9-15,47-57,59,60,82].

The assessment was independently conducted by two reviewers, with disagreements resolved by discussion. The PICOTS framework definition and domain-specific ratings are provided in Multimedia Appendix 2.

## Results

### Overview of Included Studies

**Figure 1.**
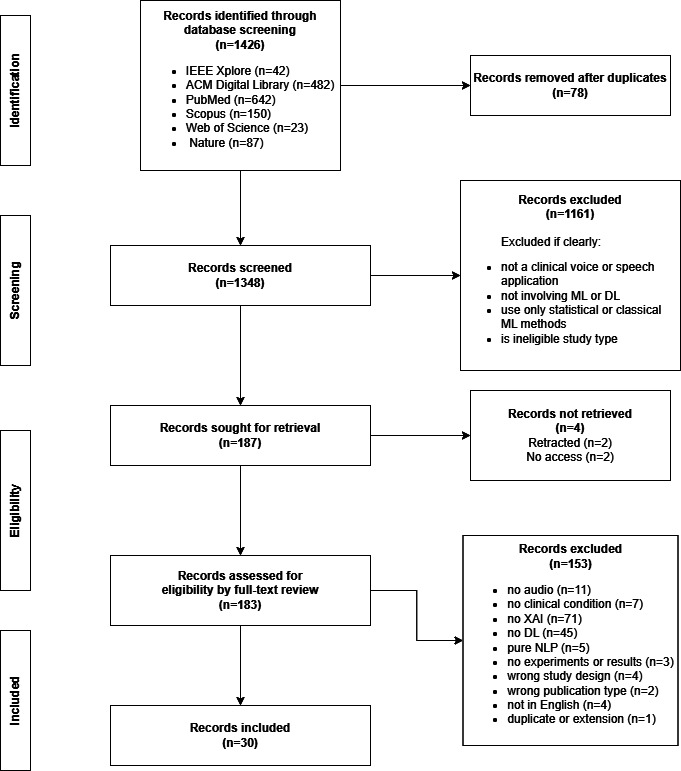
PRISMA flowchart. DL: deep learning; ML: machine learning; NLP: natural language processing; PRISMA: Preferred Reporting Items for Systematic Reviews and Meta-Analyses; XAI: explainable artificial intelligence.

A total of 30 studies met the inclusion and exclusion criteria. Although the search spanned publications from 2015 to 2025, all included studies were published from 2020 onward, except for a single paper. This can be explained by the relatively recent adoption of deep learning-based approaches and XAI for voice and speech in health care research. A PRISMA flow diagram summarizing the selection process is shown in Figure 1. The results are summarized in Table 2.

**Table 2.** Overview of the 30 included studies applying explainable and interpretable deep learning methods to clinical voice and speech analysis. The table summarizes clinical application domains, datasets, modeling approaches, and explainability techniques. Across studies, post hoc, local explainability methods predominated, with latent representation analysis, most commonly t-SNE^a^, being the most frequently used approach. Overall, explanations were primarily interpreted qualitatively, with limited quantitative validation, external consistency assessment, or human-centered evaluation reported.

Work	Application	Model	Model performance	XAI^b^ method	XAI insight	Quantitative XAI output validation
Shaikh et al [4]	Voice disorder classification	MLP^c^, 1D-CNN^d^	97.1% accuracy, 99.8% recall, 97% F1	Saliency maps	Top five LLD^e^ features were extracted per condition; saliency maps emphasized low amplitude, high-frequency spectrogram regions, though no consistent pattern emerged.	Cross-dataset evaluation
Gupta et al [50]	Multiclass dysarthria severity classification	ResNet-14^f^	98.8% accuracy, 98.9% F1	Guided backpropagation	For low-severity cases, the model focused on high-energy vowel regions (clearer phoneme boundaries); for high-severity cases, activations were diffuse, consistent with phonatory instability and temporal smearing.	—^g^
Fu et al [48]	Schizophrenia vs healthy (binary)	Sch-Net (CNN^h^ with skip connections, CBAM^i^)	97.68% accuracy, 99.1% recall, 97.7% F1	Grad-CAM^j^	Reduced high-frequency energy focus (*<*5 kHz) and emphasis on low-frequency formant stripes (<2 kHz), suggesting articulation errors (voiced instead of unvoiced consonants) aligned with blunted affect.	—
Lee et al [6]	Postoperative vocal recovery (GRBAS^k^)	EfficientNet-B4 (CNN)+ LSTM^l^	0.379 RMSE^m^ (regression), 91.8% AUC^n^ (breathiness, binary)	Grad-CAM	Different activation patterns by GRBAS level: attention to low bands (0‐2 kHz, formants), mid bands (2‐4 kHz, harmonics/noise), and temporal regions (pauses/breathy segments).	—
Peng et al [3]	Multiclass voice disorder classification	OpenL3+ SVM^o^	99.5% accuracy, 99.6% recall, 99.6% F1	Grad-CAM, t-SNE	Disorder-specific band focus: healthy in low-frequency regions; hyperkinetic dysphonia in both high and low bands; reflux laryngitis in high bands; hypokinetic dysphonia weak across bands.	—
Rojas et al [47]	Mild traumatic brain injury detection	ResNet	67.4% accuracy, 76.1% recall, 69.9% F1	Grad-CAM	Model down-weighted low frequencies and emphasized high-frequency regions for mTBI^p^ predictions.	—
Shen and Zhang [49]	Speech disfluency detection	Multiadversarial neural network	58.7% UAR^q^	Time-related Grad-CAM, t-SNE	Highlighted frames aligned with annotated disfluencies; distinct disfluency types exhibited different temporal Grad-CAM patterns; results supported wav2vec capturing meaningful temporal cues.	—
Jeong et al [10]	Parkinson disease classification	AST^r^, EfficientNet-based	92.15% accuracy, 91.5% recall, 92.15% F1	Eigen-CAM^s^	Emphasis on higher-frequency bands associated with muffled/degraded speech in PD^t^.	Annotation-based verification
Schultebraucks et al [51]	PTSD^u^/major depressive disorder (binary and severity) at 1 month	Multimodal DNN^v^	90% AUC, 84% recall, 83 F1 (PTSD), 86% AUC, 82% recall, 82% F1 (depression)	SHAP^w^	Key predictors: reduced pitch/intensity (voice), negative affect/self-focus (language), flat affect (face).	—
Ditthapron et al [53]	TBI^x^ vs healthy (binary)	pSinc+ cGRU^y^	83.8% balanced accuracy, 92.9% recall, 85.1% F1	GradientSHAP^z^, Sinc filters	High attribution to filler words and high-frequency spectral patterns; formants were salient for TBI detection.	—
Zhang et al [9]	Dementia detection	BiLSTM^aa^+ multihead attention (audio); DistilBERT^ab^+1D-CNN+ cross-modal attention	80.8% accuracy, 77.57% recall, 83.23% F1 (external validation)	SHAP (LLDs), LIME^ac^ (text/audio)	Linguistic features (eg, noun phrase rate, word rate) were most predictive among LLDs; AD^ad^ speech showed more fillers/pronouns/function words, lower energy, and slower rate; attention emphasized disfluent/low-energy segments.	—
Gutiérrez-Serafín et al [54]	Brain lesion detection	CNN	73% accuracy, 75% recall, 75% F1	xDMFCCs^ae^ (LIME)	MFCC^af^-1/2 (energy/clarity) were most important; controls showed earlier/clearer articulation with more discriminative energy in higher-order MFCCs; patients showed delayed onset, slower articulation, and longer phoneme duration.	—
Liu et al [18]	Automatic dysarthric speech recognition	TDNN-HMM^ag^; CTC^ah^; LAS^ai^, encoder–decoder	25.5% WER^aj^	Ablation studies	TDNN^ak^ outperformed CTC on moderate-severe dysarthria (highlighting the importance of temporal dependencies); speaker adaptation substantially reduced WER (per-speaker customization is beneficial).	—
Huang et al [14]	Schizophrenia severity (classification/regression)	Transformer embeddings (BERT^al^, ELECTRA^am^, TERA^an^)+ BiLSTM + FC^ao^	88% accuracy, 80% F1 (severity classification)	Ablation studies	BERT is most crucial for the TLC^ap^ scale and for PANSSs^aq^ except PANSS-General; ELECTRA contributed moderately; TERA was low for TLC but important for PANSS, especially positive symptoms and general psychopathology.	—
Herath et al [82]	Aphasia severity classification	DNN	98.5% accuracy, 97.3% recall, 97.4% F1	Ablation studies	MFCC-DNN performed best; ZCR-DNN^ar^ performed worst.	—
He et al [55]	Schizophrenia detection	WNSA-Net^as^	98.16% accuracy, 98.72% F1 (TORGO^at^)	Ablation studies	Wideband and narrowband spectrograms provided complementary information; dilated convolutions captured micro-level (pitch, formants) and macro-level (rate, prosody) cues.	Cross-dataset evaluation
Lahoti et al [11]	Parkinson disease detection	Multihead attention BiLSTM	85.02% accuracy, 84.9% F1	Ablation studies	Augmenting cepstral features with shifted delta cepstra improved performance over single-frequency filtering cepstral coefficients alone, highlighting long-term temporal dependencies for PD detection.	—
Zhang et al [15]	Depression detection	Wav2vec+1D-CNN+ LSTM	90.9% accuracy, 90.7% F1, 95.6% AUC (binary)	Ablation studies	Models with self-attention performed better; wav2vec embeddings were superior; 7-second segments worked best, suggesting emotion is concentrated in short spans.	—
Laguarta and Subirana [12]	Alzheimer disease detection	Open voice brain model (GNN^au^)	93.3% accuracy, 95% AUC	Ablation studies	Memory/fluency features dominated early-stage AD detection, with sentiment/prosody also contributing; AD often showed high saliency in respiratory control, disfluency, or memory-related patterns.	—
Joshy and Rajan [56]	Dysarthria severity classification	DNN, CNN, GRU^av^	93.97% accuracy (speaker dependent), 70.52% accuracy (speaker independent)	Ablation studies; t-SNE	MFCCs performed best in a speaker-dependent setup; CQCCs^aw^ generalized better to unseen speakers; articulatory features were strongest among disorder-specific sets but weaker in a speaker-independent setup; MFCC-based i-vectors showed clearer class clustering in t-SNE.	Multidataset evaluation
Yue et al [20]	Automatic dysarthric speech recognition	Multistream CNN + LiGRU^ax^	30.3% WER (dysarthric), 11% WER (typical)	Ablation; CNN filter analysis; t-SNE	Best WER resulted from combining spectrogram magnitude with vocal tract and excitation streams; speed perturbation without F0 fixing improved WER; filters fed with vocal-tract signals emphasized low quefrencies, whereas excitation filters suppressed them; t-SNE showed progressive dysarthric/typical separation and reduced gender clustering over training.	—
Wang et al [52]	Auditory verbal hallucination detection	Uni-modal BiGRU^ay^; multimodal self-attention DNN	84% F1 (overall), 78% F1 (audio, text)	Simple attention visualization, SHAP	Attention prioritized clauses describing distress, influence, or interference, aligning higher weights with higher auditory verbal hallucination severity.	—
Lau et al [57]	Voice disorder detection	AST	81.9% UAR, 91.1% AUC	t-SNE, attention rollout	Model focused on specific phonemes (eg, /ɔ/ and the segment “/e/ /s/ /i/ /n/”) rather than merely high-energy regions.	—
Abderrazek et al [58]	Head and neck cancer intelligibility	CNN	0.91 PCC^az^	Concept detector network	No neurons detected phonetic features in the first dense layer; from subsequent layers to output, the number of phonetic feature detectors increased by a factor of 1.75.	Cross-dataset evaluation
Mathad et al [7]	Hypernasality assessment (children with cleft palate)	DNN	0.797 PCC	Concept detector network	A DNN nasality model estimated posterior probabilities for nasal consonants, oral consonants, nasalized vowels, and oral vowels; these were combined into an objective hypernasality measure that quantified detected nasality against expected nasality per phrase.	Cross-dataset evaluation
Hung et al [5]	Voice disorder classification	SincNet (CNN-based)	83.3% accuracy, 77.31% UAR	Sinc filter analysis, t-SNE	SincNet filters emphasized F1/F2 more clearly than standard CNN filters, preserving formant structure and energy in 500‐3000 Hz bands.	—
Vasquez-Correa et al [13]	Parkinson disease assessment	CNN	97.6% accuracy, 98.7% AUC (multimodal)	Feature map analysis	Feature maps showed filters highlighting speech transitions (syllable onsets/offsets); many filters in layers 2 and 4 differentiated PD from healthy controls.	—
Lee et al [60]	ASD^ba^ detection in infants	BiLSTM with autoencoder	68.18% accuracy, 65.1% UAR, 54.57% F1	t-SNE	Autoencoder embeddings yielded clearer ASD vs TD^bb^ separation than eGeMAPS^bc^ with BiLSTM.	—
Geng et al [19]	Automatic dysarthric speech recognition	TDNN; conformer	25.5% WER	t-SNE	SVD^bd^-based spectrotemporal deep embeddings showed better separation of dysarthric vs typical speech than i-vectors/x-vectors.	Cross-language, multidataset evaluation
Kim et al [59]	Laryngeal disease classification	ResNet-50	92.15% accuracy, 91.53% recall, 92.15% F1	t-SNE	Pooled CNN features for benign disease overlapped with cancer and vocal cord paralysis, explaining reduced multiclass performance.	—

a t-SNE: t-distributed stochastic neighbor embedding.

b XAI: explainable artificial intelligence.

c MLP: multilayer perceptron.

d 1D-CNN: one-dimensional convolutional neural network.

e LLD: low-level descriptor.

f ResNet-14: residual network.

g Not available.

h CNN: convolutional neural network.

i CBAM: convolutional block attention module.

j Grad-CAM: gradient-weighted class activation mapping.

k GRBAS: Grade, Roughness, Breathiness, Asthenia, Strain.

l LSTM: long short-term memory.

m RMSE: root mean squared error.

n AUC: area under the curve.

o SVM: support vector machine.

p mTBI: mild traumatic brain injury.

q UAR: unweighted average recall.

r AST: audio spectrogram transformer.

s Eigen-CAM: Eigen class activation mapping.

t PD: Parkinson disease.

u PTSD: posttraumatic stress disorder.

v DNN: deep neural network.

w SHAP: Shapley Additive Explanations.

x TBI: traumatic brain injury.

y cGRU: cascading gated recurrent unit.

z GradientSHAP: gradient Shapley Additive Explanations.

aa BiLSTM: bidirectional long short-term memory.

ab DistilBERT: distilled version of Bidirectional Encoder Representations from Transformers.

ac LIME: local interpretable model-agnostic explanations.

ad AD: Alzheimer disease.

ae xDMFCC: explainable deep learning mel-frequency cepstral coefficients.

af MFCC: mel-frequency cepstral coefficient.

ag TDNN-HMM: time-delay neural network—hidden Markov model

ah CTC: connectionist temporal classification.

ai LAS: Listen, Attend, and Spell model architecture.

aj WER: word error rate.

ak TDNN: time delay neural network.

al BERT: Bidirectional Encoder Representations From Transformer.

am ELECTRA: Efficiently Learning an Encoder That Classifies Token Replacements Accurately.

an TERA: Transformer Encoder Representations From Alteration.

ao FC: fully connected layer.

ap TLC: Thought, Language, and Communication.

aq PNASS: Positive and Negative Syndrome Scale.

ar ZCR-DNN: deep neural network with zero-crossing rate features as input.

as WNSA-Net: axial-attention-based network using wideband and narrowband spectrograms.

at TORGO: database of acoustic and articulatory speech from speakers with dysarthria (University of Toronto).

au GNN: graph neural network.

av GRU: gated recurrent unit.

aw CQCC: constant Q cepstral coefficients.

ax LiGRU: light gated recurrent unit.

ay BiGRU: bidirectional gated recurrent unit.

az PCC: Pearson correlation coefficient.

ba ASD: autism spectrum disorder.

bb TD: typically developing.

bc eGeMAPS: extended Geneva Minimalistic Acoustic Parameter Set.

bd SVD: Saarbrücken Voice Database.

The included studies applied explainability methods across a broad range of voice- and speech-related health domains, including voice and structural laryngeal pathology [3-6,58,59], Parkinson disease (PD) [10,11,13], dysarthria and automatic dysarthric speech recognition [18-20,50,51], dementia [9] and Alzheimer disease (AD) detection [12], psychiatric and mental health conditions [15,48,52,55], traumatic brain injury (TBI) and focal brain lesions [47,53,54], aphasia [82], cleft palate-related hypernasality assessment [7], head and neck cancer–related intelligibility assessment [58], and autism spectrum disorder [60].

Datasets used across the included studies varied in quality, scale, language, and provenance. Most datasets were collected in controlled clinical environments, with only a single study [52] relying on remotely collected speech data. Dataset sizes ranged from approximately 15 participants to several hundred. Table 3 reports dataset sizes and tasks reported in their respective data collection protocols, not the studies included in this literature review. The datasets used represent a range of linguistic backgrounds, with English, Chinese, and Korean datasets being the most prevalent. Publicly available benchmark datasets were predominantly used in studies focusing on neurodegenerative diseases and motor speech disorders, whereas studies targeting psychiatric conditions relied exclusively on institution-specific or private datasets (eg, TORGO, UASpeech (Universal Access Speech), and PC-GITA [Parkinson Corpus – Grupo de Investigación en Telecomunicaciones Aplicadas]). Speech elicitation tasks varied across studies and included sustained vowel phonation, diadochokinesis, read speech, picture description, and free or spontaneous speech.

**Table 3.** Overview of speech and voice datasets used in the reviewed studies, including clinical domain, language, participant population, elicited speech tasks, and study usage. The datasets encompass a wide range of domains, sizes, and languages. UASpeech^a^ was the most frequently used publicly available benchmark, while other studies relied on their own data collection protocols.

Dataset name	Application/domain	Language	Population	Tasks	Used by
ADReSS^b^ (DementiaBank) [83]	Alzheimer disease	English	156 subjects (78 AD^c^, 78 controls)	Cookie theft picture	[9,12]
Pitt Corpus (DementiaBank) [84]	Alzheimer disease	English	500 (253 AD, 247 controls)	Cookie theft picture, word fluency task, spontaneous interviews	[9]
AVH^d^ Voice Diaries Dataset [52]	Auditory verbal hallucinations	English	384 participants	30-day audio diary recordings	[52]
SNUBH^e^ Infant Dataset [60]	Autism	Korean	39 infants (10 ASD^f^, 29 TD^g^)	Clinical vocalizations during ASD assessment	[60]
LANNA^h^ Speech Corpus [85]	Specific language impairment	Czech	188 children (118 SLI^i^, 70 controls)	Vowels, consonants, syllables, words, sentences, picture description	[48]
Max-Planck Brain Lesion Dataset [54]	Brain lesions	Dutch	16 patients with lesions, 16 controls	Emotion-elicited word production	[54]
Americleft Database [86]	Cleft palate	English	60 children with CP^j^, 10 controls	Sentences	[7]
NMCPC^k^ Database [87]	Cleft palate	English	32 children with CP, 9 controls	Sentences	[7]
JCCOCC^l^ MoCA^m^ Cantonese Speech corpus [88]	Cognitive impairment	Cantonese	469 speakers	Cognitive assessment interviews	[19]
Bellevue Trauma Dataset [89]	Depression or PTSD^n^	English, Spanish, and Mandarin	377 (first round) 221 (second round)	Clinical interviews	[51]
CMDC^o^ [90]	Depression	Cantonese	78 speakers	Structured interviews (audio, video, text)	[15]
DAIC-WOZ^p^ [91]	Depression, PTSD, anxiety	English	189 speakers	Semistructured clinical interviews (audio, video, text)	[15]
CUDYS^q^ Corpus [92]	Dysarthria	Cantonese	27 dysarthric speakers	Short sentence recordings	[18]
TORGO^r^ [93]	Dysarthria	English	15 speakers (7 dysarthric, 7 controls)	Nonwords, words, sentences	[20,56]
UASpeech^a^ [94]	Dysarthria	English	29 speakers (16 dysarthric, 13 controls)	Isolated words	[18-20,50,56]
KSoF^s^ [95]	Fluency/stuttering	German	37 speakers	Therapy-based speech recordings	[49]
SEP-28k-E^t^ [96]	Fluency/stuttering	English	21,857 three-second clips (23 h)	Spontaneous speech (podcasts)	[49]
BREF^u^ [97]	Healthy speech	French	≈120 speakers (100 h)	Newspaper reading	[58]
C2SI-LEC^v^ [98]	Head and neck cancer	French	94 patients, 41 controls	Pseudo-words, image description, read speech	[58]
DIRAMS^w^ dataset [6]	Thyroidectomy speech impairment	Korean	114 patients (preoperation, 2-wk postoperation, 3 mo postoperation)	1‐20 s utterance	[6]
PC-GITA^x^ [99]	PD^y^	Spanish	100 subjects (50 patients with PD, 50 controls)	Sustained vowel, DDK^z^, words, sentences, read passage, free speech	[11,13]
Sangmyung University PD Dataset [10]	PD	Korean	200 speakers (100 PD, 100 controls)	Vowels, consonants, DDK	[10]
Ruhr University PD Dataset [100]	PD	German	168 idiopathic PDs	DDK, read passage	[13]
CzechPD [101]	PD	Czech	46 speakers (23 PD, 23 controls)	Sustained vowel, DDK, read passage, free speech	[13]
Sichuan University Schizophrenia Dataset [48]	Schizophrenia	Mandarin	28 patients, 28 controls	Emotion-elicited reading	[48]
NTUH^aa^ Schizophrenia Dataset [14]	Schizophrenia	Taiwanese	26 patients	Clinical interviews	[14]
Coelho Corpus [102]	TBI^ab^	English	55 TBI, 52 controls	Memory task, picture description	[53]
Adolescent mTBI^ac^ Dataset [47]	mTBI	English	72 concussion, 93 controls	Multisyllabic word reading	[47]
FEMH^ad^ Speech Disorder Database [103]	Voice disorders	Mandarin	1061 samples (101 neoplasm, 100 functional dysphonia, 124 vocal palsy, 718 phonotrauma, 100 normal)	Sustained vowel	[5]
Saarbrücken Voice Database [104]	Voice disorders	German	687 healthy, 1356 patients	Sustained vowels, pitch glides, read phrase	[4,57]
VOICED^ae^ [105]	Voice disorders	Italian	208 samples (150 pathological, 58 healthy)	Sustained vowel	[3,4]
Yeouido St. Mary Hospital of the Catholic University Voice Dataset [59]	Voice disorder	Korean	30 laryngeal cancer, 97 vocal fold paralysis, 81 benign mucosal disease, 155 controls	Sustained vowel	[59]

a UASpeech: Universal Access Speech.

b ADReSS: Alzheimer's Dementia Recognition Through Spontaneous Speech.

c AD: Alzheimer disease.

d AVH: auditory verbal hallucination.

e SNUBH: Seoul National University Bundang Hospital.

f ASD: autism spectrum disorder.

g TD: typically developing.

h LANNA: Laboratory of Artificial Neural Network Applications.

i SLI: specific language impairment.

j CP: cleft palate.

k NMCPC: New Mexico Cleft Palate Center.

l JCCOCC: Jockey Club Centre for Osteoporosis Care and Control.

m MoCA: Montreal Cognitive Assessment.

n PTSD: posttraumatic stress disorder.

o CMDC: Chinese Multimodal Depression Corpus.

p DAIC-WOZ: Distress Analysis Interview Corpus – Wizard of Oz.

q CUDYS: Chinese University of Hong Kong Dysarthric Speech.

r TORGO: database of acoustic and articulatory speech from speakers with dysarthria (University of Toronto).

s KSoF: Kassel State of Fluency.

t SEP-28k-E: Stuttering Events in Podcasts (extended).

u BREF: a large read-speech corpus for French (Computer Science Laboratory for Mechanics and Engineering Sciences – French National Centre for Scientific Research).

v C2SI-LEC: Carcinologic Speech Severity Index corpus – short text reading task.

w DIRAMS: Dongnam Institute of Radiological and Medical Sciences.

x PC-GITA: Parkinson Corpus – Grupo de Investigación en Telecomunicaciones Aplicadas.

y PD: Parkinson disease.

z DDK: diadochokinesis.

aa NTUH: National Taiwan University Hospital.

ab TBI: traumatic brain injury.

ac mTBI: mild traumatic brain injury.

ad FEMH: Far Eastern Memorial Hospital.

ae VOICED: Voice Icarfederico II.

### Explainability Methods

Explainability methods used in the included literature are summarized in Table 1. Across the 30 included studies, gradient-based saliency methods, input perturbation techniques, and model-internal representation analysis were the most frequently used explainability approaches.

Gradient-based saliency methods were reported in 7 studies [3,4,6,47-50], with Grad-CAM being the most commonly used technique, appearing in 5 studies. Grad-CAM [106] is a model-specific technique that uses gradients of convolutional feature maps to generate class-discriminative explanations as coarse (ie, non–pixel-level) heatmaps. Other gradient-based approaches, including guided backpropagation and vanilla saliency maps, were rarely used [4,50]. Vanilla saliency [107] computes pixel-level importance by calculating gradients of the model output (ie, logits) with respect to the input features, where the magnitude of the gradient at each feature indicates its importance to the model’s decision. Guided backpropagation [108] differs from vanilla saliency maps in that gradients are only propagated when both the forward activation and the backward gradient are positive, resulting in cleaner, less noisy, and more visually interpretable maps. Unlike Grad-CAM, vanilla saliency and guided backpropagation operate directly at the input level and do not provide explicit, spatially localized class-discriminative explanations.

Input perturbation-based techniques were also widely adopted across applications. SHAP was implemented in 4 studies spanning dementia detection [9], auditory verbal hallucination assessment [52], psychiatric disorder classification [51], and TBI [53]. SHAP [109] is a model-agnostic explainability method grounded in cooperative game theory that attributes to each feature a contribution score, quantifying how the presence or absence of that feature influences a model’s prediction. Ablation-based analysis was the most prevalent perturbation method, reported in 9 studies, and was applied across multiple clinical tasks [11,12,14,15,18,20,55,56,82]. Ablation studies usually involve systematic removal of specific parts of the model or input features and observing the resulting impact on model performance.

Model-internal representation analysis was reported in 12 studies, where interpretability was derived through inspection or visualization of learned internal model representations. Although primarily used as a dimensionality reduction technique, t-distributed stochastic neighbor embedding (t-SNE) [110] dominated this category, appearing in 9 studies [3,5,19,20,49,56,57,59,60], for its utility in mapping high-dimensional latent representations into low-dimensional space while preserving local neighborhood relationships. Additional representation-level analysis included inspection of learned convolutional filters [13,20], parameterized filter structures (ie, Sinc filters) [5], and dimensionality reduction of feature maps (ie, Eigen class activation maps [Eigen-CAM]) [10], providing insight into how internal model components responded to speech signals. Sinc filters are parameterized to directly model frequency bands, allowing them to more effectively emphasize formant frequencies. As Sinc filters effectively function as bandpass filters, they offer conceptually more interpretable representations compared to the abstract patterns learned by arbitrarily shaped CNN filters [5]. Unlike Grad-CAM, Eigen-CAM [111] is a gradient-free method that calculates saliency maps by taking the first principal component of flattened feature maps.

Surrogate model-based methods were less frequently used among the included literature [9,54]. Most notably, LIME [112] is a model-agnostic explainability method that learns a simple, interpretable surrogate model, such as linear models or decision trees, that locally approximates the predictions of a black-box model. An adaptation of LIME, explainable deep learning mel-frequency cepstral coefficients (xDMFCC), is another surrogate-based model designed for interpreting MFCC-based audio representations [54].

Concept-based methods, which link internal model activations to predefined, human-interpretable concepts to explain predictions in domain-relevant terms, were implemented in 2 studies [7,58].

Lastly, attention-based explanations were reported in 2 studies, where interpretability was derived either through direct visualization of attention weights [113] or through attention rollout-based aggregation of attention across layers [57].

### Explainability Input-Output Representation

Reported explainability methods operated over a range of input representations, which ultimately defined the form and interpretability of the resulting explanations.

Time-frequency and cepstral representations, including wideband and narrowband spectrograms, mel-spectrograms, and MFCCs, were commonly interrogated by gradient-based saliency methods [3,4,6,10,47,49,50]. In these cases, explanations were presented as heatmaps highlighting salient temporal segments and spectral bins of the input representation.

Surrogate-based methods and input perturbation techniques typically operate over cepstral representations and acoustic low-level descriptors (LLDs) [9,51]. These approaches produced explanations in the form of numerical feature importance values and ranking scores. Various audio representations were used for ablation analysis, where interpretable insight was based on tabular performance changes, following feature removal or modification [11,12,14,15,18,20,55,56,82].

Model-internal representation analyses, such as t-SNE, derived interpretability from latent representations learned by deep models, with explanations expressed as 2D projections revealing structure or separation in the latent space [3,5,19,20,49,56,57,59,60]. In a small number of studies, parameterized filter models operating directly on raw waveforms were used, where interpretability was provided through filter frequency responses identifying salient frequency bands [5,53].

Finally, concept-based approaches operated on frame-level cepstral representations, where neural activations were inspected in relation to predefined, human-interpretable semantic or clinically meaningful concepts [7,58].

### Domain-Specific Explanation Patterns

#### Overview

Outputs of explainability and interpretability methods were synthesized according to the target clinical application, thus identifying recurring clinical interpretability themes across the literature for each outcome domain.

#### Voice and Structural Laryngeal Pathology

Explanations within the voice disorder classification and laryngeal pathology literature indicate saliency across low- to midfrequency regions associated with formant structure and harmonic organization, as well as high-frequency components indicative of noise and phonatory instability. For example, for voice disorders classification, gradient-based methods emphasized low-amplitude, high-frequency spectrotemporal regions [4], while disorder-specific band emphasis patterns were reported in the study by Peng et al [3]. On a similar note, t-SNE analysis for pooled CNN features revealed overlap for benign and malignant laryngeal diseases [59]. On the other hand, parameterized Sinc filters were found to more explicitly capture F1 and F2 frequencies and energy bands compared to traditional CNN filters [5]. Postoperative recovery assessment using GRBAS (Grade, Roughness, Breathiness, Asthenia, Strain) scores showed distinct activation patterns within the 0‐2 kHz region typical of formant structure, the 2‐4 kHz band associated with harmonics and noise components, and temporally localized regions corresponding to pauses and breathiness [6]. In head and neck cancer, articulatory attributes were found to be important for intelligibility assessment through the discovery of progressively increasing phonetic feature detectors (neurons) across deeper layers of the concept detector network [58]. Ultimately, explanations consistently conveyed the importance of phonatory control, harmonic structure, and articulatory clarity as commonly highlighted cues.

While the studies by Peng et al [3] and Shaikh et al [4] relied on the VOICED (Voice Icarfederico II) dataset, other studies based their investigations on distinct public or locally collected datasets. Thus, the aforementioned explanation patterns do not seem to be confined to a single dataset.

#### Parkinson Disease

Insights drawn from explainability analysis revealed degraded spectral patterns and impaired articulatory transitions consistent with hypokinetic dysarthria. Eigen-CAM emphasized higher frequencies typical of muffled and degraded speech [10]. CNN filter analysis indicated slower and less distinct phoneme transitions for patients with PD [13]. Similarly, ablation studies underscored the importance of long-term temporal dependencies and cepstral dynamics demonstrating the altered articulatory dynamics of patients with PD [11].

It is worth noting that the studies by Lahoti et al [11] and Vasquez-Correa et al [13] used the same dataset (PC-GITA); therefore, convergent findings between these 2 studies should be interpreted with appropriate context.

#### Dysarthria and Dysarthric Speech Recognition

Ablation analyses in dysarthria severity classification and automatic dysarthric speech recognition applications identified various spectral and temporal features as significant contributors to model decisions. Guided backpropagation revealed diffuse and less localized activation patterns in high-severity dysarthric cases, in contrast to more focused vowel-centered saliency in milder cases [50]. In ASR models, ablation and latent representation analyses emphasized the importance of temporal dependency modeling through time delay neural network architectures [18], speaker adaptation mechanisms [20], and low-quefrency components reflecting vocal tract characteristics [19]. Additionally, MFCC-, constant Q cepstral coefficients–, and articulatory-based feature sets demonstrated varying performance across speaker-dependent and speaker-independent ASR systems [56]. Notably, the findings across these studies do not constitute independent validation, as all dysarthria-focused experiments were conducted using the same dataset (UASpeech), as shown in Table 3. Therefore, these findings are data-specific and are not generalizable conclusions.

#### Dementia and Alzheimer Disease

In dementia detection, SHAP and LIME analysis identified noun phrase rate, empty word rate, and hesitation ratio as influential linguistic and fluency features indicative of decreased lexical complexity and increased disfluency in AD speech, alongside voice-related features reflecting lower vocal energy [9]. In addition to linguistic attributes, graph-based models in the study by Laguarta and Subirana [12] highlighted memory-related biomarkers in early-stage AD. It is important to note that both studies used the ADReSS (Alzheimer's Dementia Recognition Through Spontaneous Speech) dataset; therefore, their convergent findings should be interpreted within the context of the same dataset.

#### Psychiatric Disorders

Explainability and interpretability analyses across psychiatric disorders were found to emphasize the importance of vocal-spectral attributes, linguistic content, and temporally localized affective states. Grad-CAM heatmaps revealed reduced high-frequency energy in schizophrenic speech and highlighted altered formant contours compared to controls, typical of articulatory disruption [48]. Ablation analysis in the study by He et al [55] showed that wideband spectrograms, capturing transient events and articulatory changes, and narrowband spectrograms, emphasizing pitch variation and voice quality, provided complementary representations for schizophrenia detection. SHAP analyses of multimodal models identified reduced voice intensity alongside linguistic markers of negative affect and self-focus as important predictors for posttraumatic stress disorder and major depressive disorder [51]. Similarly, voice quality and prosodic features ranked among the top SHAP predictors for auditory verbal hallucination detection, while attention heatmaps prioritized textual clauses reflecting distress and interference [52]. In the study by Zhang et al [15], self-attention mechanisms applied to shorter audio segments enhanced depression detection, suggesting the temporal locality of depressive state-related cues. Across psychiatric applications, explanations frequently reflected prosodic flattening, altered spectral energy distribution, and content-level emotional markers.

#### Traumatic Brain Injury and Brain Lesion Detection

In TBI, GradientSHAP identified high-frequency spectral patterns and filler words as salient markers [53], and Grad-CAM highlighted high-frequency components in mild traumatic brain injury classification [47]. xDMFCC analysis demonstrated the importance of lower-order cepstral coefficients as indicators of spectral sharpness and speech clarity in brain lesion detection [54]. In contrast, higher-order cepstral coefficients did not clearly capture phoneme transitions in patients. The study concluded that brain lesion speech was characterized by delayed onset, slower articulation, and prolonged phoneme duration. Explanations across these studies allude to reduced articulatory precision and spectral clarity as discriminatory markers.

#### Other Clinical Domains

Several additional clinical applications were represented by single studies and therefore did not permit cross-study pattern synthesis.

The work by Mathad et al [7] performed a hypernasality assessment using a concept detector network to estimate posterior probabilities of nasal and oral phoneme classes, which were subsequently combined into an objective hypernasality measure. Similarly, Abderrazek et al [58] used a concept detector framework for head and neck cancer intelligibility assessment, using a French phone classifier to identify phonetic feature detectors (ie, internal neurons) that informed a quantitative intelligibility metric. Ablation analysis in the study by Herath et al [82] demonstrated the superiority of MFCCs over alternative spectral and temporal representations for aphasia severity classification. For autism spectrum disorder detection, t-SNE plots revealed clearer separation of autoencoder embeddings compared to handcrafted acoustic features [60]. Finally, in the study by Shen and Zhang [49], they applied time-related Grad-CAM to highlight temporally localized activations aligned with manually-annotated stutter disfluency segments.

#### Explainability Validation Strategies

Of the 30 included studies, 6 performed model-centric explainability validation using external data [4,7,19,55,56,58], including 1 study [19] that explicitly assessed the cross-language consistency of explanation patterns. Ground-truth or annotation-based verification was reported in a single study [49], where salient temporal regions identified by the explainability method were compared against reference labels provided by nonclinical annotators.

The remaining studies did not conduct quantitative validation of explainability outputs. Instead, 10 studies relied solely on qualitative interpretation of explanations and comparison with findings reported in prior literature [9,10,12,13,48,50-52,54,57]. In these cases, explanations were assessed narratively for plausibility or consistency with known clinical or acoustic characteristics without formal evaluation of faithfulness, robustness, or stability. Zhang et al [9] and Fu et al [48] performed cross-dataset evaluation not as consistency analysis for explainability but as external validation for the underlying model. Lastly, 12 studies reported explainability outputs without conducting quantitative analysis or qualitative literature comparisons [3,5,6,11,14,15,18,20,53,59,60,82].

#### Human-Centered Analysis and Stakeholder Alignment

None of the included studies explicitly reported formal human-centered evaluation of explainability outputs, such as structured assessment of explanations by clinicians, speech-language pathologists, or regulatory stakeholders.

To contextualize potential stakeholder involvement, we additionally recorded the domain expertise of study authors. Approximately half of the included studies [3,4,9,11,12,15,18-20,49,50,53,55,56,82] were authored exclusively by technical researchers (eg, computer scientists, engineers, or biomedical engineers), while the remaining studies included at least one author with nontechnical domain expertise (eg, clinical, medical, or speech-language pathology background). However, the presence of nontechnical coauthors did not correspond to explicit reporting of human-in-the-loop evaluation of explainability outputs.

#### Quality and Risk of Bias Assessment

A total of 25 studies were eligible for PROBAST+AI assessment, while 5 studies were not assessed because their models did not predict health-related outcomes. Further, 3 of these studies developed automatic dysarthric speech recognition models, and 2 studies developed models predicting phonetic features. Although the outputs of these models were later used to derive clinical measures, the models themselves did not constitute health-related prediction tasks within the scope of PROBAST+AI.

For model development, 80% (20/25) of studies were judged to have an overall high-quality concern, 16% (4/25) of studies had an overall low-quality concern, and 4% (1/25) of studies received an overall unclear rating. Domain 4 (analysis) was the primary driver of great concern, followed by domain 1 (participants and data sources). In domain 4, concerns were mainly related to small development datasets relative to model complexity and insufficient safeguards against overfitting. In domain 1, concerns stemmed from unclear or restrictive inclusion and exclusion criteria, limiting representativeness. Domains 2 (predictors) and 3 (outcome) were rated as low concern in nearly all studies. Applicability concerns across domains 1‐3 were rated as low for all assessed studies.

For model evaluation, 92% (23/25) of studies were judged to have a high risk of bias, 4% (1/25) of studies a low risk, and 4% (1/25) of studies unclear risk. As in development, domains 4 and 1 were the main contributors to elevated risk. Evaluation splits were frequently small and unlikely to be representative, particularly in relation to model complexity. Data leakage was identified in 6 studies. Furthermore, performance assessment rarely extended beyond standard discrimination metrics; calibration assessment and decision-analytic measures were largely absent. Domains 2 and 3 were rated as low risk of bias across all studies. Detailed domain-level ratings for each study are provided in Multimedia Appendix 2.

## Discussion

### Methodological Quality of Underlying Prediction Models

The concentration of quality concerns within the analysis domain, as identified by PROBAST+AI [80], has important implications for XAI applications and the interpretability of explanations. The use of complex, high-capacity deep learning models trained and evaluated on limited clinical voice datasets, often without external validation or calibration assessment, increases the risk of overfitting to dataset-specific characteristics rather than generalizable, condition-relevant attributes.

This is especially important because post hoc explainability methods are contingent on the model’s internal representations and input-output relationships. Explanations derived from overfitted or insufficiently validated models may appear coherent while reflecting confounding artifacts or spurious correlations [32,72]. Thus, the credibility of XAI methods does not rest solely on the faithfulness of the explainability technique, but also on the methodological rigor of the development and evaluation of the underlying predictive models.

These considerations are particularly relevant for the analysis of the domain-specific explanation patterns. Although recurring themes were observed across the clinical domains, the risk of bias of the underlying models and circular validation across studies due to the use of the same dataset should motivate the reader to view the findings or explanations with caution, as they may reflect data-specific characteristics rather than generalizable disease-related insights. Accordingly, the purpose of presenting domain-specific explanation patterns is not to establish definitive clinical explanatory signatures, but rather to characterize the current landscape of explainability practice in voice and speech AI and to motivate more rigorous, validated, and clinically grounded approaches in future work.

### Interpretation of Explanations

Although current XAI methods provide preliminary insight into the inner workings of clinical audio models, the interpretation of explainability outputs is rarely subjected to rigorous validation. Many studies rely on a limited number of illustrative examples to interpret local explanations (eg, saliency maps or attention weights) without quantitative assessment or statistical analysis [3,4,6,48,50,57]. This increases the risk of overinterpretation such that visually compelling or anecdotal explanations are inferred to be clinically meaningful despite limited evidence of generalizability.

Some studies attempt to contextualize explanation outputs by comparison with established clinical or acoustic knowledge [9,10,12,48,50-52,54]. While such comparisons may enhance face validity, they do not guarantee faithfulness of the explanation to the model’s true decision process. Prior work has demonstrated, for example, that attention weights are not inherently faithful indicators of feature importance and may exhibit weak or inconsistent correspondence with gradient-based relevance measures, leading to potentially misleading interpretations [114-116]. Interpreting explanations primarily through the lens of existing medical literature may therefore introduce confirmation bias, whereby explanations that align with prior expectations are accepted uncritically while alternative patterns are overlooked [35].

These concerns are reinforced by the fact that most reviewed studies [3,6,9,10,12,15,18,47,48,50-54] did not perform any form of quantitative explainability validation. In the absence of systematic evaluation, explanatory interpretations are more vulnerable to confirmation bias and may overstate their clinical relevance. This pattern might be indicative of underlying issues relevant to clinical voice and speech research. The scarcity of qualitative explanation validation is possibly driven by the high cost and, subsequently, the scarcity of annotated, high-quality clinical voice and speech datasets. The same reason might also explain the high risk of bias for most included studies, even for otherwise methodologically sound ones.

### Complexity-Transparency Trade-Off

The reviewed studies suggest an inherent trade-off between model complexity, input representation, and explainability in clinical audio-based AI systems [32]. In particular, the interpretability of model explanations is closely linked to the degree of semantic transparency in the input features. Audio representation in the literature included raw audio [49], low-level acoustic descriptors (eg, jitter, shimmer, and harmonic-to-noise ratio) [51,60], raw time-frequency representations (eg, spectrograms and mel-spectrograms) [6,48,50,82], coefficient-based features (eg, MFCCs) [7,11,56,82], and deep neural network–transformer-based embeddings (eg, i-vectors, wav2vec, and HuBERT [Hidden-Unit Bidirectional Encoder Representations From Transformers]) [14,18,19,60].

Models trained on manually extracted LLDs offer the highest degree of interpretability, as these features are directly related to established clinical biomarkers or constructs. Explanations from models trained on LLDs can be relatively easily understood using feature attribution methods such as SHAP or LIME. This alignment between model explanations and clinical knowledge contributes to the popularity of such methods in the medical AI domain.

While MFCCs are also hand-engineered features, they present interpretability challenges due to their abstract and decorrelated nature [117]. The coefficients do not correspond directly to intuitive phonetic or physiological phenomena. Although recent techniques such as xDMFCC attempt to provide coefficient-level explanations across time, and Tracey et al [117] sought to demystify MFCCs as vocal biomarkers by correlating them with known LLDs, the clinical relevance of these explanations remains limited. Despite efforts to position MFCCs as vocal biomarkers, they largely obscure the internal workings of deep models and contribute to the opacity of clinical audio systems [118].

While raw audio exhibits energy and temporal information, spectrograms offer a more interpretable alternative. As visual time-frequency representations, they capture dynamic changes in energy that are often physically and clinically meaningful, such as vocal formants or pauses. Trained speech pathologists and clinicians can interpret spectrograms directly, making visualization-based explanations (eg, saliency maps) more accessible and potentially clinically actionable compared to MFCCs. Although visual explanations of spectrograms might inform about the time segment most important for the detection of a condition and can highlight relevant frequency bands, more granular visual explanations are typically not feasible.

Finally, state-of-the-art systems increasingly rely on transformer-based embeddings such as wav2vec and HuBERT, which yield task-optimized, high-dimensional representations learned from raw waveforms. While these embeddings deliver substantial performance gains, they are the most difficult to interpret due to high-dimensional, nonlinear abstraction from both low-level acoustic features and clinically grounded descriptors. As a result, explanations tend to be less granular, less transparent, and harder to align with clinical reasoning.

### Misalignment of Explanations With Stakeholder Needs

**Figure 2.**
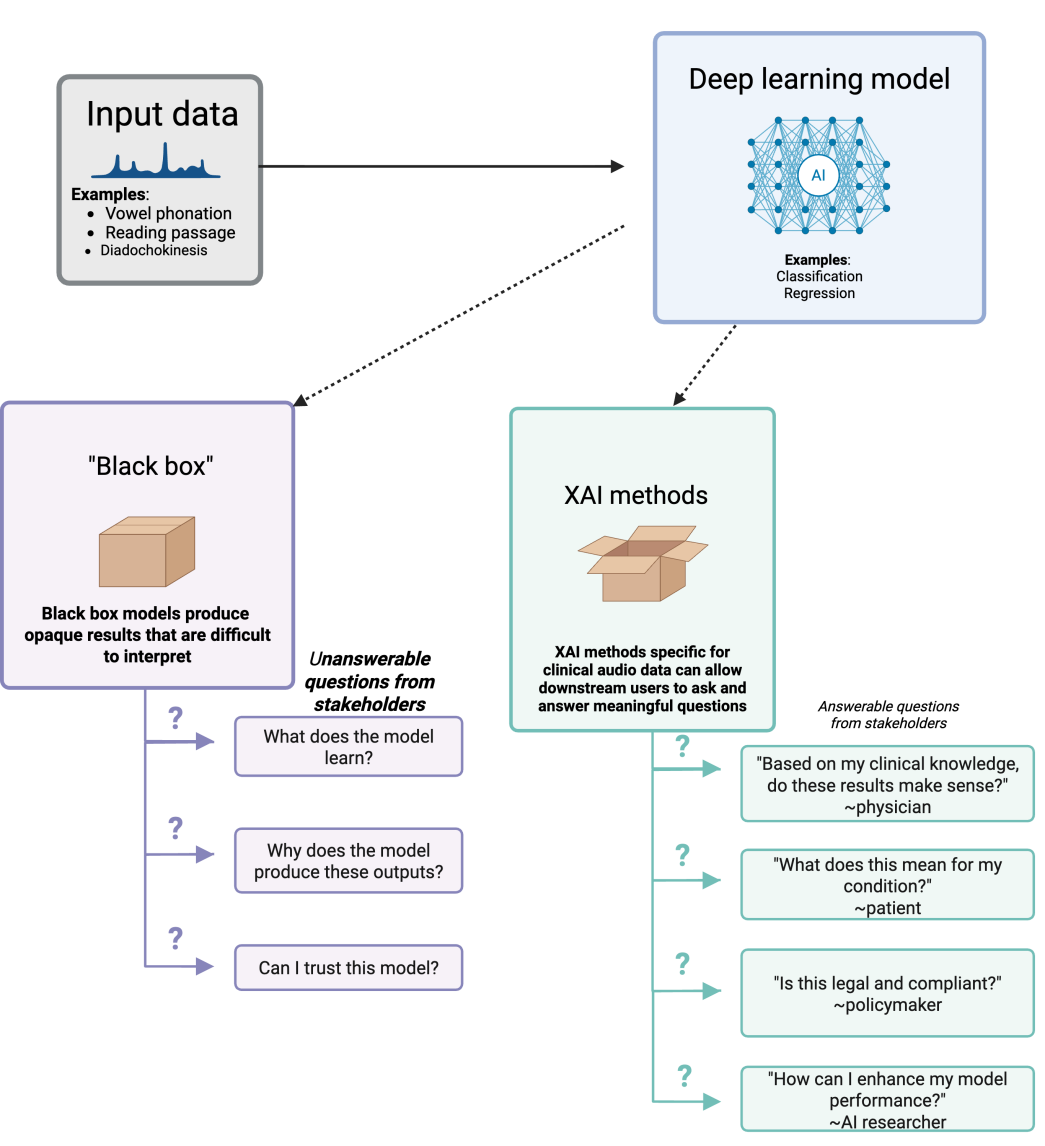
Although XAI aims to address the black-box issue of deep learning models, current XAI methods do not cater to the diverse expectations and needs of the different stakeholders. AI: artificial intelligence; XAI: explainable artificial intelligence.

A major theme in the literature is the misalignment of the explanation form or modality and the needs of the end users or stakeholders. All included studies produce explanations targeted for highly technical audiences (ie, AI researchers and developers), without taking into consideration the interpretive frameworks of clinicians, patients, regulators, and policymakers. While such explanations offer insight to AI developers and researchers, critical for understanding model behavior, enhancing performance, and ensuring reliability, these explanations are often too technical, abstract, or detached from domain-specific language to be directly actionable in clinical decision-making. Figure 2 highlights the central challenge of stakeholder–explanation misalignment in clinical voice and speech audio AI, illustrating how diverse stakeholders hold distinct expectations and informational needs, and emphasizing the necessity of tailoring explanation strategies accordingly.

Explanations that focus on algorithmic mechanisms poorly convey information in terms of established diagnostic criteria or clinical reasoning processes [35]. Patients, with their highly limited technical and clinical knowledge, are less likely to draw relevant insights from such technical explanation modalities such as activation maps and feature attribution plots [119]. Similarly, regulators and policymakers prefer explanations that offer transparent, auditable decision pathways to assess compliance, fairness, and accountability [120].

This gap is partly a result of explanation design being driven primarily by XAI method availability rather than user requirements [121]. Most of the reviewed literature adapts generic explainability techniques to clinical audio tasks without taking into consideration the information needs, domain expertise, or cognitive constraints of their target users. This highlights the imperative need for human-centered, context-aware design of clinical audio explainability methods in which concerned stakeholders participate and provide valuable input for suitable explanation modalities and content.

### Future Directions

**Figure 3.**
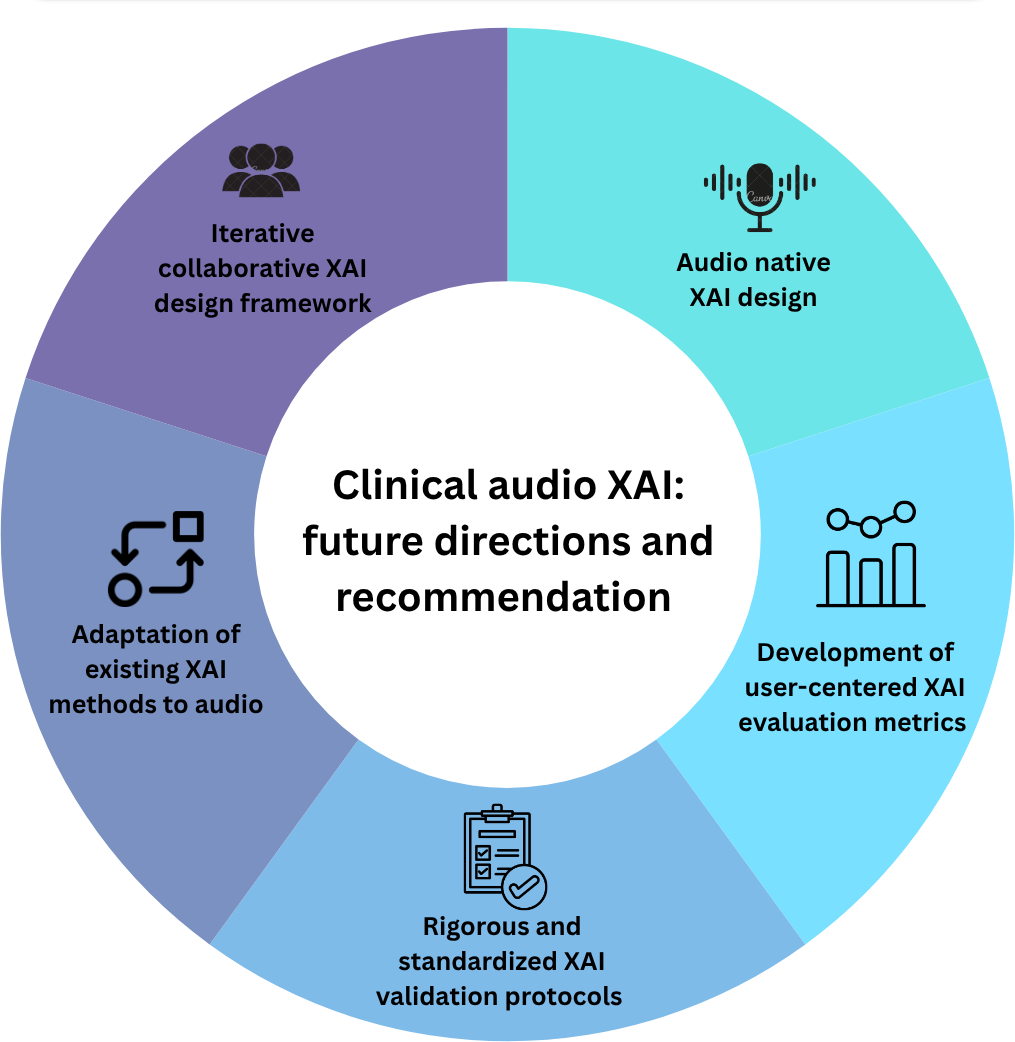
The future of voice and speech XAI in health care lies in the integration of perceptually aligned explanation methods, robust evaluation frameworks, and stakeholder-centered design, enabling explanations that are both faithful to model behavior and meaningful in real-world clinical practice. XAI: explainable artificial intelligence.

In this work, we discussed several limitations of current methods and identified opportunities for advancing interpretability and explainability in clinical audio-based deep learning systems. Accordingly, this section outlines future directions and recommendations for advancing XAI specifically for clinical audio applications, as summarized in Figure 3.

Although the literature used various explainability strategies, several techniques that are well established in related domains have rarely been adapted for clinical audio-based systems. For instance, example-based and counterexample-based explainability methods are widely used in audio-based emotion recognition [73] to generate sonified explanations. Similarly, in the text-to-audio domain, AudioGenX quantifies the importance of textual tokens corresponding to generated audio using factual and counterfactual techniques [16]. Integrating these approaches into clinical voice and speech analysis can enhance explanation fidelity and stakeholder interpretability.

Moreover, many of the XAI techniques surveyed in this review are originally developed for image or tabular data, underscoring the need for domain-specific approaches tailored to the temporal-spectral nature of audio signals and the heterogeneous manifestations of speech and voice disorders. This is especially important given the perceptual nature of audio. These methods should aim to map abstract representations (eg, deep transformer-based embeddings) to established clinical constructs, thereby bridging the gap between model outputs and actionable clinical insight. For example, CoughLIME (Cough Local Interpretable Model-Agnostic Explanations) [122] extended LIME to generate sonified explanations for COVID-19 cough analysis. Future work should aim to develop sonified XAI that aligns with the perceptive ability of clinicians (eg, speech-language pathologists). Recent progress in large audio language models is garnering interest due to chain-of-thought reasoning and their capability to identify environmental sounds, speech characteristics, and respiratory and heart sounds [123,124]. These models can be used to extract salient acoustic features and present them in structured, report-style textual summaries through a stakeholder-friendly interface.

Our review also identified a lack of rigorous validation of XAI explanations. Future work should use both quantitative measures (eg, fidelity, sensitivity, perturbation-based testing, and cross-dataset explanation consistency) and qualitative, human-centered evaluation strategies (eg, expert annotation comparison and interrater agreement). For example, a recent work introduces a frequency band perturbation framework for quantitatively evaluating the faithfulness of various XAI techniques [125].

Additionally, the misalignment of explanation formats with stakeholder needs highlights the importance of iterative, collaborative design processes in which AI developers engage clinicians, patients, and regulators throughout system development. Such collaboration can help ensure that explanations are understandable, clinically relevant, and operationally feasible, while supporting auditability, transparency, and accountability. For instance, the work by Pizzimenti et al [126] describes a Delphi study aimed at unifying and standardizing vocal biomarker research where clinicians, statisticians, audio signal processing experts, AI researchers, and ethicists are involved in this endeavor. Such efforts highlight the importance of structured, interdisciplinary validation frameworks for clinical audio research.

Finally, throughout this systematic review, we encountered substantial difficulty in quantifying the degree of explainability and practical utility of reported methods, particularly across different stakeholder groups. Consistent with prior findings [44], only a small number of studies formally evaluated the effectiveness of XAI in clinical settings. Future work should therefore develop composite evaluation frameworks that integrate objective indicators (eg, performance improvement and error detection) with subjective measures (eg, perceived clarity, trust, and usability), enabling comparison of explainability methods in terms of both model alignment and real-world clinical impact. This is achieved by addressing limitations identified under PROBAST+AI, such that future work prioritizes study designs with representative cohorts, sufficient sample sizes, and appropriate model evaluation of diagnostic and prognostic modeling. Such practices will ensure the development of methodologically sound models, such that clinical outcomes derived from explainability methods are of high quality and clinical value, thus increasing the trustworthiness of AI in clinical practice.

### Limitations

This review has several limitations that should be considered when interpreting the findings. Study selection and data extraction were conducted by a single reviewer. Although established PRISMA procedures and predefined inclusion criteria were followed, the absence of a second independent screening may increase the risk of selection bias or missed studies.

Second, conclusions regarding domain-specific explanation patterns are constrained by the methodological quality of the underlying prediction models. A substantial proportion of studies exhibited a high risk of bias, particularly in model development and evaluation. In addition, several studies relied on repeated use of the same benchmark datasets across dysarthria, PD, and AD domains, raising the risk of circular validation and inflating apparent consistency of explanation patterns. The domain-specific synthesis presented in this review is therefore intended to characterize current practice rather than establish definitive clinical explanatory signatures.

Finally, given the rapid growth of this field, studies published after the search period (February 2025) may provide additional insights and are not reflected in this review.

### Conclusions

In this systematic review, we presented current practices of explainability and interpretability for deep learning–based voice and speech analysis in clinical care. Across 30 eligible studies, we identified a diverse set of explainability methods, which we organized into commonly adopted categories. Our findings indicate that, although explainability techniques are increasingly applied across a wide range of clinical speech and voice applications, their use is largely exploratory and rarely supported by rigorous validation. Explanations were predominantly assessed through qualitative interpretation, with limited evaluation of faithfulness, robustness, or consistency across datasets, and no explicit human-in-the-loop assessment involving clinical or regulatory stakeholders. Additionally, the quality of underlying models limits the validity of the reported explanation patterns and, subsequently, the applicability of these models for real-world clinical applications. These findings highlight the need for domain-specific, clinically grounded explainability methods, standardized validation protocols, and stakeholder-aware explanation design to support the safe and effective integration of voice and speech AI into clinical practice.

## Supplementary material

10.2196/83790 Multimedia Appendix 1 Database search queries.

10.2196/83790 Multimedia Appendix 2 PROBAST+AI risk assessment.

10.2196/83790 Checklist 1 PRISMA checklist.

## Abbreviations

AD: Alzheimer disease
ADReSS: Alzheimer's Dementia Recognition Through Spontaneous Speech
AI: artificial intelligence
ASR: automatic speech recognition
CNN: convolutional neural network
CoughLIME: Cough Local Interpretable Model-Agnostic Explanations
CT: computed tomography
ECG: electrocardiogram
EHR: electronic health record
Eigen-CAM: Eigen class activation maps
Grad-CAM: gradient-weighted class activation mapping
GRBAS: Grade, Roughness, Breathiness, Asthenia, Strain (voice scale)
HuBERT: Hidden-Unit Bidirectional Encoder Representations from Transformers
LIME: local interpretable model-agnostic explanations
LLD: low-level descriptor
MFCC: mel-frequency cepstral coefficients
ML: machine learning
MRI: magnetic resonance imaging
PC-GITA: Parkinson Corpus – Grupo de Investigación en Telecomunicaciones Aplicadas
PD: Parkinson disease
PICOTS: Population; Index Model; Comparator; Outcome; Timing; Setting
PRISMA: Preferred Reporting Items for Systematic Reviews and Meta-Analyses
PROBAST+AI: Prediction Model Risk of Bias Assessment Tool for Artificial Intelligence
SHAP: Shapley Additive Explanations
t-SNE: t-distributed stochastic neighbor embedding
TBI: traumatic brain injury
UASpeech: Universal Access Speech
VOICED: Voice Icarfederico II
XAI: explainable artificial intelligence
xDMFCC: explainable deep learning mel-frequency cepstral coefficients
