# Supplementary material for: Explainable and Interpretable AI for Voice and Speech Analysis in Clinical Care: Systematic Review
Source: J Med Internet Res. 2026 Jun 24;28:e83790. doi: 10.2196/83790 (PMC13293602; doi:10.2196/83790)
Supplement: Multimedia Appendix 1 — Database search queries. [file jmir-v28-e83790-s001.docx]

**Scopus**
( TITLE-ABS-KEY ( "Explainable AI" OR "interpretable AI" OR "XAI" OR "explainable machine learning" OR "interpretable machine learning" OR "feature attribution" OR "model interpretability" OR "post-hoc explainability" OR "SHAP" OR "LIME" OR "Grad-CAM" OR "attention" OR "saliency maps" OR "Layer-wise Relevance Propagation" OR "LRP" ) AND TITLE-ABS-KEY ( "voice biomarker" OR "speech biomarker" OR "speech analysis" OR "acoustic analysis" OR "phonetic analysis" OR "speech processing" OR "vocal assessment" ) AND TITLE-ABS-KEY ( "healthcare" OR "medical diagnosis" OR "neurological disorder" OR "neurodegenerative disease" OR "mental health" OR "biomarker" OR "voice disorder" OR "speech disorder" OR "voice quality" OR "vocal quality" OR "clinical voice analysis" OR "disordered speech" ) ) AND ( PUBYEAR > 2014 AND PUBYEAR < 2026 ) AND ( LIMIT-TO ( LANGUAGE , "English" ) )

**IEEE Xplore**

("All Metadata":"Explainable AI" OR "All Metadata":"interpretable AI" OR "All Metadata":"XAI" OR "All Metadata":"explainable machine learning" OR "All Metadata":"interpretable machine learning" OR "All Metadata":"feature attribution" OR "All Metadata":"model interpretability" OR "All Metadata":"post-hoc explainability" OR "All Metadata":"SHAP" OR "All Metadata":"LIME" OR "All Metadata":"Grad-CAM" OR "All Metadata":"attention" OR "All Metadata":"saliency maps" OR "All Metadata":"Layer-wise Relevance Propagation" OR "All Metadata":"LRP") AND ("All Metadata":"voice biomarker" OR "All Metadata":"speech biomarker" OR "All Metadata":"speech analysis" OR "All Metadata":"acoustic analysis" OR "All Metadata":"phonetic analysis" OR "All Metadata":"speech processing" OR "All Metadata":"vocal assessment") AND ("All Metadata":"healthcare" OR "All Metadata":"medical diagnosis" OR "All Metadata":"neurological disorder" OR "All Metadata":"neurodegenerative disease" OR "All Metadata":"mental health" OR "All Metadata":"biomarker" OR "All Metadata":"voice disorder" OR "All Metadata":"speech disorder" OR "All Metadata":"voice quality" OR "All Metadata":"vocal quality" OR "All Metadata":"clinical voice analysis" OR "All Metadata":"disordered speech")

**PubMed**

("Artificial Intelligence"[MeSH] OR "Machine Learning"[MeSH] OR "Neural Networks, Computer"[MeSH] OR "Decision Support Techniques"[MeSH] OR "Pattern Recognition, Automated"[MeSH] OR "Natural Language Processing"[MeSH] OR "Deep Learning" OR "Explainable AI" OR "interpretable AI" OR "XAI" OR "explainable machine learning" OR "interpretable machine learning" OR "feature attribution" OR "model interpretability" OR "post-hoc explainability" OR "SHAP" OR "LIME" OR "Grad-CAM" OR "attention" OR "saliency maps" OR "Layer-wise Relevance Propagation" OR "LRP") AND ("Voice"[MeSH] OR "Speech"[MeSH] OR "Speech Acoustics"[MeSH] OR "Speech Production Measurement"[MeSH] OR "Phonetics"[MeSH] OR "Voice Quality"[MeSH] OR "speech biomarker" OR "voice biomarker" OR "speech analysis" OR "acoustic analysis" OR "phonetic analysis" OR "speech processing" OR "vocal assessment") AND ("Neurological Disorders"[MeSH] OR "Parkinson Disease"[MeSH] OR "Alzheimer Disease"[MeSH] OR "Mental Disorders"[MeSH] OR "Biomarkers"[MeSH] OR "Voice Disorders"[MeSH] OR "Speech Disorders"[MeSH] OR "clinical voice analysis" OR "disordered speech")

**Nature**

("Explainable AI" OR "interpretable AI" OR "XAI" OR "explainable machine learning" OR "interpretable machine learning" OR "feature attribution" OR "model interpretability" OR "post-hoc explainability" OR "SHAP" OR "LIME" OR "Grad-CAM" OR "attention" OR "saliency maps" OR "Layer-wise Relevance Propagation" OR "LRP") AND ("voice biomarker" OR "speech biomarker" OR "speech analysis" OR "acoustic analysis" OR "phonetic analysis" OR "speech processing" OR "vocal assessment") AND ("healthcare" OR "medical diagnosis" OR "neurological disorder" OR "neurodegenerative disease" OR "mental health" OR "biomarker" OR "voice disorder" OR "speech disorder" OR "voice quality" OR "vocal quality" OR "clinical voice analysis" OR "disordered speech")

**Web of Science**

TS=("Explainable AI" OR "interpretable AI" OR "XAI" OR "explainable machine learning" OR "interpretable machine learning" OR "feature attribution" OR "model interpretability" OR "post-hoc explainability" OR "SHAP" OR "LIME" OR "Grad-CAM" OR "attention" OR "saliency maps" OR "Layer-wise Relevance Propagation" OR "LRP") AND TS=("voice biomarker" OR "speech biomarker" OR "speech analysis" OR "acoustic analysis" OR "phonetic analysis" OR "speech processing" OR "vocal assessment") AND TS=("healthcare" OR "medical diagnosis" OR "neurological disorder" OR "neurodegenerative disease" OR "mental health" OR "biomarker" OR "voice disorder" OR "speech disorder" OR "voice quality" OR "vocal quality" OR "clinical voice analysis" OR "disordered speech") AND PY=(2015-2025)

**ACM**

[[All: "explainable ai"] OR [All: "interpretable ai"] OR [All: "xai"] OR [All: "explainable machine learning"] OR [All: "interpretable machine learning"] OR [All: "feature attribution"] OR [All: "model interpretability"] OR [All: "post-hoc explainability"] OR [All: "shap"] OR [All: "lime"] OR [All: "grad-cam"] OR [All: "attention"] OR [All: "saliency maps"] OR [All: "layer-wise relevance propagation"] OR [All: "lrp"]] AND [[All: "voice biomarker"] OR [All: "speech biomarker"] OR [All: "speech analysis"] OR [All: "acoustic analysis"] OR [All: "phonetic analysis"] OR [All: "speech processing"] OR [All: "vocal assessment"]] AND [[All: "healthcare"] OR [All: "medical diagnosis"] OR [All: "neurological disorder"] OR [All: "neurodegenerative disease"] OR [All: "mental health"] OR [All: "biomarker"] OR [All: "voice disorder"] OR [All: "speech disorder"] OR [All: "voice quality"] OR [All: "vocal quality"] OR [All: "clinical voice analysis"] OR [All: "disordered speech"]] AND [E-Publication Date: (01/01/2015 TO 02/11/2025)]

| Number of results | Data Source/Search Engine | Filters |
| --- | --- | --- |
| 150 | Scopus | Filters: Year, Language |
| 42 | IEEE | Year, conferences, journals |
| 642 | PubMed | Year |
| 87 | Nature | Year |
| 23 | Web of Science | Year |
| 482 | ACM | Year, Conferences, and journals |
