# Supplementary material for: Explainable and Interpretable AI for Voice and Speech Analysis in Clinical Care: Systematic Review
Source: J Med Internet Res. 2026 Jun 24;28:e83790. doi: 10.2196/83790 (PMC13293602; doi:10.2196/83790)
Supplement: Multimedia Appendix 2 — PROBAST+AI risk assessment. [file jmir-v28-e83790-s002.docx]

**PICOTS Framework**

- **Population (P):**
  Human participants represented in clinical or health-related speech and/or voice datasets used for the development or evaluation of prediction models. Studies may include participants with and without clinical conditions; population characteristics are assessed at the individual study level for representativeness and applicability.
- **Index model(s) (I):**
  **Deep learning–based prediction models** that use speech or voice data as input to estimate a clinical or health-related outcome. The prediction model itself is the object of risk-of-bias assessment; explainability methods applied to these models are considered ancillary components.
- **Comparator(s) (C):**
  Not prespecified at the review level. When reported, alternative deep learning architectures, classical machine-learning models, baseline methods, or human comparators are extracted descriptively but are not required for inclusion or risk-of-bias assessment.
- **Outcome(s) (O):**
  Study-defined clinical or health-related outcomes predicted by the model, such as diagnostic status, disease severity, symptom presence, or clinical classification labels. Outcome definitions and reference standards are evaluated at the individual study level.
- **Timing (T):**
  The intended time point of prediction is at the moment the speech or voice sample is recorded and available for model input. The prediction horizon is typically cross-sectional or immediate, unless a study explicitly specifies a prognostic time horizon.
- **Setting and intended use (S):**
  Clinical and health-related research settings in which speech or voice data are collected for prediction purposes, including clinical environments, research cohorts, and curated datasets. Intended use may include screening, diagnosis, assessment, or monitoring.

**PROBAST+AI Domain Ratings for Model Development**

Study-level PROBAST+AI domain ratings for model development. Q denotes quality concern and A denotes applicability concern. Domains were rated as low, high, or unclear in accordance with PROBAST+AI guidance. Overall ratings were classified as high if at least one domain was rated high. Applicability was assessed for the first three domains only and not for the Analysis domain. The fourth domain does not include applicability considerations under the PROBAST+AI framework, as applicability refers to the assessor’s review question or intended use of a model, including the target population and setting.

| **Work** | **Domain 1: Participants & Data** | | **Domain 2: Predictors** | | **Domain 3: Outcome** | | **Domain 4: Analysis** | **Overall Judgement** | |
| --- | --- | --- | --- | --- | --- | --- | --- | --- | --- |
|  | **Q** | **A** | **Q** | **A** | **Q** | **A** | **Q** | **Q** | **A** |
| Shaikh et al. [4] | LOW | LOW | LOW | LOW | LOW | LOW | HIGH | HIGH | LOW |
| Gupta et al. [71] | HIGH | LOW | LOW | LOW | LOW | LOW | HIGH | HIGH | LOW |
| Fu et al. [73] | LOW | LOW | LOW | LOW | LOW | LOW | HIGH | HIGH | LOW |
| Lee et al. [6] | LOW | LOW | LOW | LOW | LOW | LOW | HIGH | HIGH | LOW |
| Peng et al. [3] | LOW | LOW | LOW | LOW | LOW | LOW | HIGH | HIGH | LOW |
| Rojas et al. [61] | HIGH | LOW | LOW | LOW | LOW | LOW | HIGH | HIGH | LOW |
| Shen and Zhang [80] | LOW | LOW | LOW | LOW | LOW | LOW | LOW | LOW | LOW |
| Jeong et al. [10] | LOW | LOW | LOW | LOW | LOW | LOW | LOW | LOW | LOW |
| Schultebraucks et al. [72] | LOW | LOW | LOW | LOW | LOW | LOW | HIGH | HIGH | LOW |
| Ditthapron et al. [76] | LOW | LOW | LOW | LOW | LOW | LOW | UNCLEAR | UNCLEAR | LOW |
| Zhang et al. [9] | LOW | LOW | LOW | LOW | LOW | LOW | HIGH | HIGH | LOW |
| Gutiérrez-Serafíın et al. [77] | HIGH | LOW | LOW | LOW | LOW | LOW | HIGH | HIGH | LOW |
| Huang et al. [14] | LOW | LOW | LOW | LOW | LOW | LOW | HIGH | HIGH | LOW |
| Herath et al. [78] | HIGH | LOW | LOW | LOW | LOW | LOW | HIGH | HIGH | LOW |
| He et al. [74] | LOW | LOW | LOW | LOW | LOW | LOW | HIGH | HIGH | LOW |
| Lahoti et al. [11] | HIGH | LOW | LOW | LOW | LOW | LOW | HIGH | HIGH | LOW |
| Zhang et al. [15] | HIGH | LOW | LOW | LOW | LOW | LOW | HIGH | HIGH | LOW |
| Laguarta and Subirana [12] | LOW | LOW | LOW | LOW | LOW | LOW | HIGH | HIGH | LOW |
| Joshy and Rajan [81] | LOW | LOW | LOW | LOW | LOW | LOW | HIGH | HIGH | LOW |
| Wang et al. [75] | LOW | LOW | LOW | LOW | LOW | LOW | LOW | LOW | LOW |
| Lau et al. [82] | HIGH | LOW | LOW | LOW | LOW | LOW | HIGH | HIGH | LOW |
| Hung et al. [5] | LOW | LOW | LOW | LOW | LOW | LOW | LOW | LOW | LOW |
| Vásquez-Correa et al. [13] | LOW | LOW | LOW | LOW | HIGH | LOW | HIGH | HIGH | LOW |
| Lee et al. [79] | HIGH | LOW | HIGH | LOW | LOW | LOW | HIGH | HIGH | LOW |
| Kim et al. [70] | HIGH | LOW | LOW | LOW | LOW | LOW | HIGH | HIGH | LOW |

**PROBAST+AI Domain Ratings for Model Evaluation**

Study-level PROBAST+AI domain ratings for model evaluation. RoB denotes risk of bias and A denotes applicability concern. Domains were rated as low, high, or unclear in accordance with PROBAST+AI guidance. Overall ratings were classified as high if at least one domain was rated high. Applicability was assessed for the first three domains only and not for the Analysis domain. The fourth domain does not include applicability considerations under the PROBAST+AI framework, as applicability refers to the assessor’s review question or intended use of a model, including the target population and setting.

| **Work** | **Domain 1: Participants & Data** | | **Domain 2: Predictors** | | **Domain 3: Outcome** | | **Domain 4: Analysis** | **Overall Judgement** | |
| --- | --- | --- | --- | --- | --- | --- | --- | --- | --- |
|  | **RoB** | **A** | **RoB** | **A** | **RoB** | **A** | **RoB** | **RoB** | **A** |
| Shaikh et al. [4] | LOW | LOW | LOW | LOW | LOW | LOW | HIGH | HIGH | LOW |
| Gupta et al. [50] | HIGH | LOW | LOW | LOW | LOW | LOW | HIGH | HIGH | LOW |
| Fu et al. [48] | HIGH | LOW | LOW | LOW | LOW | LOW | HIGH | HIGH | LOW |
| Lee et al. [6] | HIGH | LOW | LOW | LOW | LOW | LOW | HIGH | HIGH | LOW |
| Peng et al. [3] | HIGH | LOW | LOW | LOW | LOW | LOW | HIGH | HIGH | LOW |
| Rojas et al. [47] | HIGH | LOW | LOW | LOW | LOW | LOW | HIGH | HIGH | LOW |
| Shen and Zhang [49] | LOW | LOW | LOW | LOW | LOW | LOW | HIGH | HIGH | LOW |
| Jeong et al. [10] | LOW | LOW | LOW | LOW | LOW | LOW | HIGH | HIGH | LOW |
| Schultebraucks et al. [51] | HIGH | LOW | LOW | LOW | LOW | LOW | HIGH | HIGH | LOW |
| Ditthapron et al. [53] | LOW | LOW | LOW | LOW | LOW | LOW | HIGH | HIGH | LOW |
| Zhang et al. [9] | LOW | LOW | LOW | LOW | LOW | LOW | HIGH | HIGH | LOW |
| Gutiérrez-Serafíın et al. [54] | HIGH | LOW | LOW | LOW | LOW | LOW | HIGH | HIGH | LOW |
| Huang et al. [14] | HIGH | LOW | LOW | LOW | LOW | LOW | HIGH | HIGH | LOW |
| Herath et al. [82] | LOW | LOW | LOW | LOW | LOW | LOW | HIGH | HIGH | LOW |
| He et al. [55] | LOW | LOW | LOW | LOW | LOW | LOW | HIGH | HIGH | LOW |
| Lahoti et al. [11] | HIGH | LOW | LOW | LOW | LOW | LOW | HIGH | HIGH | LOW |
| Zhang et al. [15] | HIGH | LOW | LOW | LOW | LOW | LOW | HIGH | HIGH | LOW |
| Laguarta and Subirana [12] | LOW | LOW | LOW | LOW | LOW | LOW | HIGH | HIGH | LOW |
| Joshy and Rajan [56] | LOW | LOW | LOW | LOW | LOW | LOW | HIGH | HIGH | LOW |
| Wang et al. [52] | LOW | LOW | LOW | LOW | LOW | LOW | LOW | LOW | LOW |
| Lau et al. [57] | HIGH | LOW | LOW | LOW | LOW | LOW | HIGH | HIGH | LOW |
| Hung et al. [5] | LOW | LOW | LOW | LOW | LOW | LOW | UNCLEAR | UNCLEAR | LOW |
| Vásquez-Correa et al. [13] | LOW | LOW | LOW | LOW | HIGH | LOW | HIGH | HIGH | LOW |
| Lee et al. [60] | HIGH | LOW | HIGH | LOW | LOW | LOW | HIGH | HIGH | LOW |
| Kim et al. [59] | HIGH | LOW | LOW | LOW | LOW | LOW | HIGH | HIGH | LOW |

**References**

3. Peng X, Xu H, Liu J, Wang J, He C. Voice disorder classification using convolutional neural network based on deep transfer learning. Scientific Reports. 2023;13(1):7264. doi: 10.1038/s41598-023-34461-9.

4. Shaikh AAS, Bhargavi MS, Naik GR. Unraveling the complexities of pathological voice through saliency analysis. Computers in Biology and Medicine. 2023;166:107566. doi: 10.1016/j.compbiomed.2023.107566.

5. Hung C-H, Wang S-S, Wang C-T, Fang S-H. Using SincNet for Learning Pathological Voice Disorders. Sensors. 2022;22(17):6634. doi: 10.3390/s22176634.

6. Lee JH, Lee CY, Eom JS, Pak M, Jeong HS, Son HY. Predictions for Three-Month Postoperative Vocal Recovery after Thyroid Surgery from Spectrograms with Deep Neural Network. Sensors. 2022;22(17):6387. doi: 10.3390/s22176387.

9. Zhang Z, Wang T, Hu Z, Yang LZ, Li H. DEMENTIA: A Hybrid Attention-Based Multimodal and Multi-Task Learning Framework With Expert Knowledge for Alzheimer's Disease Assessment From Speech. IEEE Journal of Biomedical and Health Informatics. 2025;29(4):2957–68. doi: 10.1109/JBHI.2024.3509620.

10. Jeong S-M, Kim S, Lee EC, Kim HJ. Exploring Spectrogram-Based Audio Classification for Parkinson’s Disease: A Study on Speech Classification and Qualitative Reliability Verification. Sensors. 2024;24(14):4625. doi: 10.3390/s24144625.

11. Lahoti A, Gurugubelli K, Arroyave JRO, Vuppala AK. Shifted Delta Cepstral Coefficients with RNN to Improve the Detection of Parkinson’s Disease from the Speech. Proceedings of the 2022 Fourteenth International Conference on Contemporary Computing; Noida, India: Association for Computing Machinery; 2022. p. 284–8. doi: 10.1145/3549206.3549258.

12. Laguarta J, Subirana B. Longitudinal Speech Biomarkers for Automated Alzheimer's Detection. Frontiers in Computer Science. 2021;3:624694. doi: 10.3389/fcomp.2021.624694.

13. Vásquez-Correa JC, Arias-Vergara T, Orozco-Arroyave JR, Eskofier B, Klucken J, Nöth E. Multimodal Assessment of Parkinson's Disease: A Deep Learning Approach. IEEE Journal of Biomedical and Health Informatics. 2019;23(4):1618-30. doi: 10.1109/JBHI.2018.2866873.

14. Huang YJ, Lin YT, Liu CC, Lee LE, Hung SH, Lo JK, et al. Assessing Schizophrenia Patients Through Linguistic and Acoustic Features Using Deep Learning Techniques. IEEE Transactions on Neural Systems and Rehabilitation Engineering. 2022;30:947–56. doi: 10.1109/TNSRE.2022.3163777.

15. Zhang X, Zhang X, Chen W, Li C, Yu C. Improving speech depression detection using transfer learning with wav2vec 2.0 in low-resource environments. Scientific Reports. 2024;14(1):9543. doi: 10.1038/s41598-024-60278-1.

47. Rojas F, Madanian S, Templeton JM, Poellabauer C, Schneider SL, editors. Exploring Deep Learning and Grad-CAM for Speech-Based Detection of Mild Traumatic Brain Injury. 2024 IEEE International Conference on Big Data (BigData); 2024. doi: 10.1109/BigData62323.2024.10825360.

48. Fu J, Yang S, He F, He L, Li Y, Zhang J, et al. Sch-net: a deep learning architecture for automatic detection of schizophrenia. BioMedical Engineering OnLine. 2021;20(1):75. doi: 10.1186/s12938-021-00915-2.

49. Shen J, Zhang X. Individual-independent and cross-language detection of speech disfluencies in stuttering based on multi-adversarial tasks and self-training. Biomedical Signal Processing and Control. 2025;100:107051. doi: 10.1016/j.bspc.2024.107051.

50. Gupta S, Patil AT, Purohit M, Parmar M, Patel M, Patil HA, et al. Residual Neural Network precisely quantifies dysarthria severity-level based on short-duration speech segments. Neural Networks. 2021;139:105–17. doi: 10.1016/j.neunet.2021.02.008.

51. Schultebraucks K, Yadav V, Shalev AY, Bonanno GA, Galatzer-Levy IR. Deep learning-based classification of posttraumatic stress disorder and depression following trauma utilizing visual and auditory markers of arousal and mood. Psychological Medicine. 2022;52(5):957–67. doi: 10.1017/S0033291720002718.

52. Wang W, Xu W, Chander A, Nepal S, Buck B, Pakhomov S, et al. The Power of Speech in the Wild: Discriminative Power of Daily Voice Diaries in Understanding Auditory Verbal Hallucinations Using Deep Learning. Proc ACM Interact Mob Wearable Ubiquitous Technol. 2023;7(3):Article 133. doi: 10.1145/3610890.

53. Ditthapron A, Lammert AC, Agu EO. Continuous TBI Monitoring From Spontaneous Speech Using Parametrized Sinc Filters and a Cascading GRU. IEEE Journal of Biomedical and Health Informatics. 2022;26(7):3517–28. doi: 10.1109/JBHI.2022.3158840.

54. Gutiérrez-Serafín B, Andreu-Perez J, Pérez-Espinosa H, Paulmann S, Ding W. Toward assessment of human voice biomarkers of brain lesions through explainable deep learning. Biomedical Signal Processing and Control. 2024;87:105457. doi: 10.1016/j.bspc.2023.105457.

55. He L, Fu J, Li Y, Xiong X, Zhang J. WNSA-Net: An Axial-Attention-Based Network for Schizophrenia Detection Using Wideband and Narrowband Spectrograms. IEEE/ACM Transactions on Audio, Speech, and Language Processing. 2023;31:721–33. doi: 10.1109/TASLP.2022.3209941.

56. Joshy AA, Rajan R. Automated Dysarthria Severity Classification: A Study on Acoustic Features and Deep Learning Techniques. IEEE Transactions on Neural Systems and Rehabilitation Engineering. 2022;30:1147-57. doi: 10.1109/TNSRE.2022.3169814.

57. Lau HS, Huntly M, Morgan N, Iyenoma A, Zeng B, Bashford T, editors. Interpreting Pretrained Speech Models for Automatic Speech Assessment of Voice Disorders. Artificial Intelligence in Healthcare; 2024; Cham: Springer Nature Switzerland. doi: 10.1007/978-3-031-67278-1_5.

59. Kim H-B, Song J, Park S, Lee YO. Classification of laryngeal diseases including laryngeal cancer, benign mucosal disease, and vocal cord paralysis by artificial intelligence using voice analysis. Scientific Reports. 2024;14(1):9297. doi: 10.1038/s41598-024-58817-x.

60. Lee JH, Lee GW, Bong G, Yoo HJ, Kim HK. Deep-Learning-Based Detection of Infants with Autism Spectrum Disorder Using Auto-Encoder Feature Representation. Sensors. 2020;20(23):6762. doi: 10.3390/s20236762.

82. Herath HMDPM, Weraniyagoda WASA, Rajapaksha RTM, Wijesekara PADSN, Sudheera KLK, Chong PHJ. Automatic Assessment of Aphasic Speech Sensed by Audio Sensors for Classification into Aphasia Severity Levels to Recommend Speech Therapies. Sensors. 2022;22(18):6966. doi: 10.3390/s22186966.
